# HiLand Resource: A Comprehensive Database of Highland Human Populations

**DOI:** 10.1093/gpbjnl/qzaf083

**Published:** 2025-09-14

**Authors:** Weijie Zhang, Xiaoning Chen, Yanling Sun, Yibo Wang, Bixia Tang, Yu Zhang, Kai Liu, Wenming Zhao, Bing Su, Yaoxi He

**Affiliations:** State Key Laboratory of Genetic Evolution & Animal Models, Kunming Institute of Zoology, Chinese Academy of Sciences, Kunming 650201, China; University of Chinese Academy of Sciences, Beijing 100049, China; University of Chinese Academy of Sciences, Beijing 100049, China; National Genomics Data Center, China National Center for Bioinformation, Beijing 100101, China; National Genomics Data Center, China National Center for Bioinformation, Beijing 100101, China; Beijing Institute of Genomics, Chinese Academy of Sciences, Beijing 100101, China; University of Chinese Academy of Sciences, Beijing 100049, China; National Genomics Data Center, China National Center for Bioinformation, Beijing 100101, China; Beijing Institute of Genomics, Chinese Academy of Sciences, Beijing 100101, China; National Genomics Data Center, China National Center for Bioinformation, Beijing 100101, China; Beijing Institute of Genomics, Chinese Academy of Sciences, Beijing 100101, China; State Key Laboratory of Genetic Evolution & Animal Models, Kunming Institute of Zoology, Chinese Academy of Sciences, Kunming 650201, China; State Key Laboratory of Genetic Evolution & Animal Models, Kunming Institute of Zoology, Chinese Academy of Sciences, Kunming 650201, China; University of Chinese Academy of Sciences, Beijing 100049, China; University of Chinese Academy of Sciences, Beijing 100049, China; National Genomics Data Center, China National Center for Bioinformation, Beijing 100101, China; Beijing Institute of Genomics, Chinese Academy of Sciences, Beijing 100101, China; State Key Laboratory of Genetic Evolution & Animal Models, Kunming Institute of Zoology, Chinese Academy of Sciences, Kunming 650201, China; Yunnan Key Laboratory of Integrative Anthropology, Kunming 650201, China; State Key Laboratory of Genetic Evolution & Animal Models, Kunming Institute of Zoology, Chinese Academy of Sciences, Kunming 650201, China; Yunnan Key Laboratory of Integrative Anthropology, Kunming 650201, China

**Keywords:** Highland, Human population, Phenotype, Genomic diversity, Genetic association

## Abstract

Over 80 million people worldwide live at high altitudes (> 2500 m), where numerous studies have documented the remarkable biological adaptations of highland populations to these extreme environments. However, current resources for accessing and analyzing highlander-specific data remain limited. To address this gap, we present the HiLand Resource (HLR), a comprehensive database that integrates phenomic, genomic, and genetic association data from 23,336 highlanders across three major high-altitude regions: the Qinghai-Tibet Plateau, the Andean Plateau, and the Ethiopian Plateau. HLR offers six key functions: (1) visualization of phenotypic patterns among highlanders from the Qinghai-Tibet Plateau across different altitudes, as well as comparison between highlanders and lowlanders, and between sexes; (2) an interactive interface to explore genomic diversity, population structure, ancestral composition, and signatures of natural selection of high-altitude populations; (3) access to a comprehensive catalog of genome-wide variants and genes identified in highlanders; (4) a genome browser built on a high-quality Tibetan genome assembly; (5) a curated collection of genotype–phenotype associations derived from genome-wide association studies (GWASs) in highland populations; and (6) an online, user-friendly tool for genotype imputation using a highland-specific reference panel. Collectively, HLR provides a novel and in-depth resource for understanding the biological features of high-altitude human populations. It holds significant potential for advancing research on human adaptation to hypoxic environments and improving medical studies focused on highland communities. The HLR database is freely available at https://ngdc.cncb.ac.cn/hiland/.

## Graphical abstract

**Figure:**
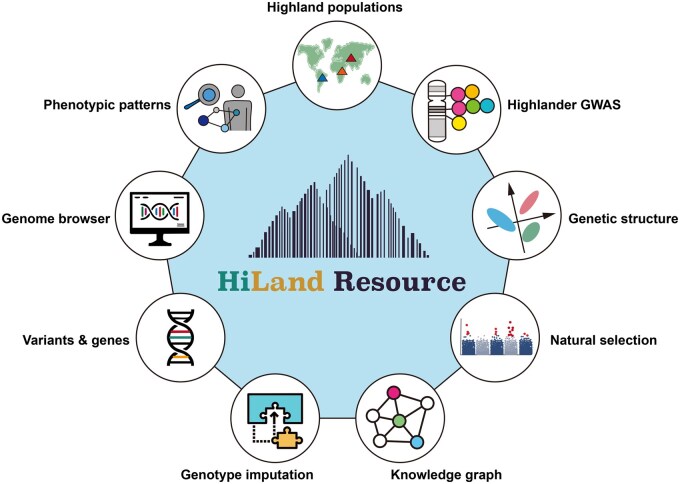

## Introduction

High-altitude environments pose significant challenges for human adaptation due to reduced barometric pressure, lower oxygen availability, and other environmental stressors such as intense ultraviolet radiation. Currently, more than 80 million people live at elevations above 2500 m [1], where blood oxygen saturation can significantly decline [2]. Three major high-altitude regions host indigenous highlander populations: the Qinghai-Tibet Plateau in Asia, the Andean Plateau in South America, and the Ethiopian Plateau in Africa. Over recent decades, numerous studies have explored the phenotypic traits and genetic adaptations of these populations [3–5]. In particular, Tibetan highlanders have been the focus of several large-scale genomic studies, including the development of a high-quality reference genome [6], whole-genome sequencing (WGS) data of 1001 individuals [7], whole-genome array (WGA) data of 3008 individuals [8], genome-wide genotyping of 2252 Tibetan mother–newborn pairs [9], and a comprehensive phenomics study involving 11,880 individuals [10]. These valuable datasets have provided new insights into the evolutionary mechanisms of high-altitude adaptation and hold promise for clinical applications to improve health outcomes in high-altitude populations.

Despite these advances, a comprehensive and integrative database that combines phenotypic, genomic, and genetic association data from diverse highland populations remains unavailable. Many existing studies suffer from limited data accessibility, hindering collaboration and in-depth analysis. Moreover, performing personalized analyses, such as visualizing genomic features, examining population structure, and conducting genotype imputation, remains challenging for high-altitude research. Although databases like *PGG.population* [11] and *PGG.Han* [12] include limited data from indigenous highlanders, they do not meet the specialized needs of high-altitude population studies.

To address these limitations, we developed the HiLand Resource (HLR), a comprehensive biological database for highlanders. HLR integrates phenotypic and genomic data from 23,336 individuals across the three major high-altitude regions: the Qinghai-Tibet Plateau, the Andean Plateau, and the Ethiopian Plateau. This database offers several key features, including global visualizations of highlander phenotypic traits, a browser for highlander reference genomes, user-friendly tools for personalized population structure analysis, and a dedicated server for highlander-specific genotype imputation. Collectively, HLR is designed to meet the diverse needs of the scientific community, enabling broader applications of genomic data in evolutionary research and high-altitude medicine.

## Database content and usage

### Overview of HLR

**Figure 1:**
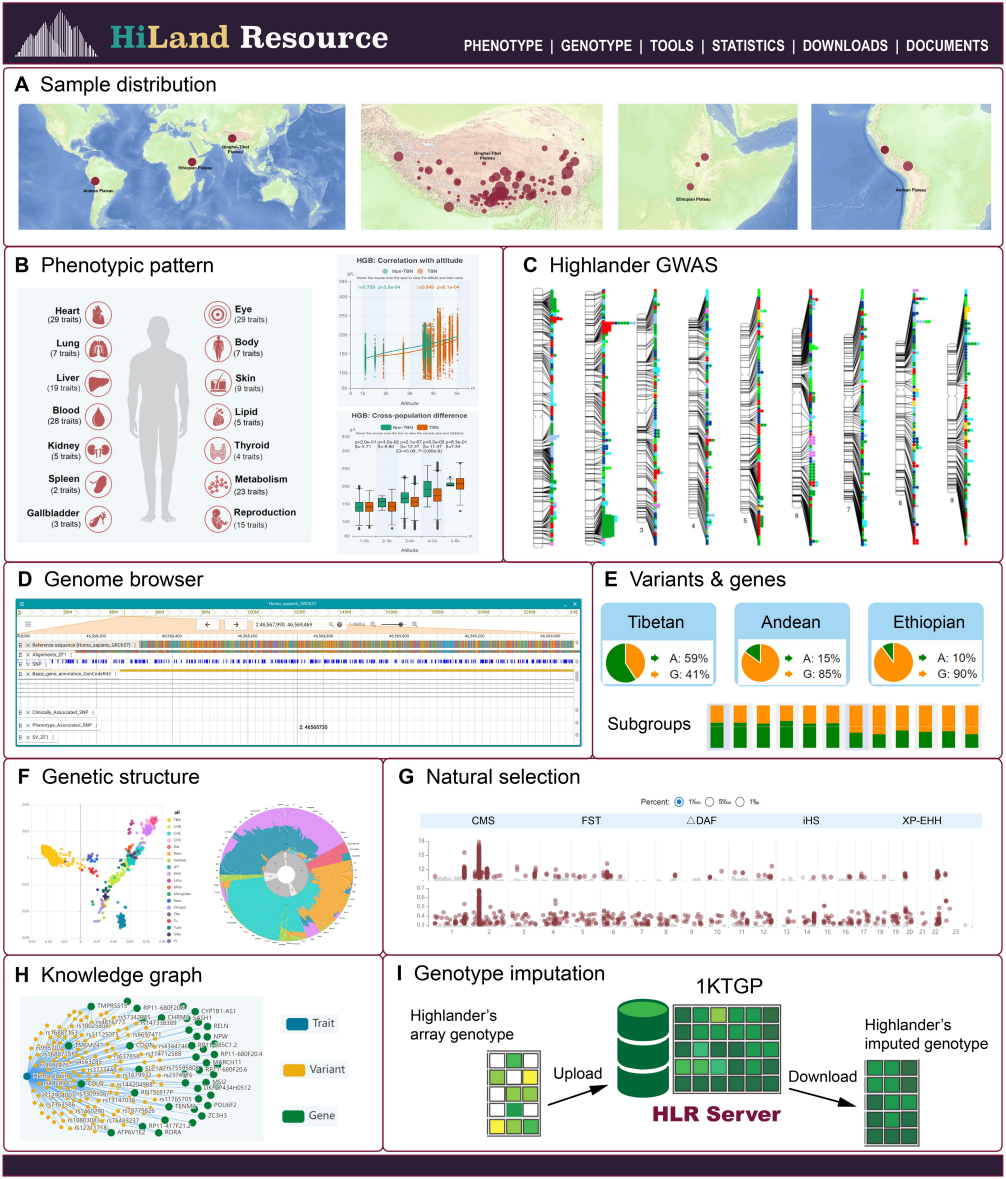
Schematic overview of HLR HLR includes ten modules, including eight visualization modules and two online analysis modules. **A**. Sample distribution. Left: global sampling map of highlanders included in this study, covering three main high-altitude areas in the world: the Qinghai-Tibet Plateau, the Andean Plateau, and the Ethiopian Plateau. Right: sampling maps of three representative highlander groups: Tibetans from the Qinghai-Tibet Plateau (left), Amharas from the Ethiopian Plateau (middle), and Quechuas from the Andean Plateau (right). **B**. Phenotypic pattern. Left: 185 traits on a human body diagram. Right: two different perspectives of HGB. **C**. Highlander GWAS showing all variant**–**trait associations reported in highlanders. Traits belong to different body system are showed in different colors. **D**. Genome browser showing the Tibetan genome ZF1 browser with multiple tracks of genomic features. **E**. Variants & genes showing an example variant with different allele frequencies in Tibetan, Andean, and Ethiopian highlanders as well as in subgroups of Tibetans. **F**. Genetic structure showing the PCA (left) and ADMIXTURE (right) results of highlanders and global populations. **G**. Natural selection showing the whole-genome scanning of positive selection signatures in the genomes of highlanders based on five methods. **H**. Knowledge graph showing the interactive association networks between genes/variants and phenotypes. **I**. Genotype imputation showing the workflow of the HLR imputation server. HLR, HiLand Resource; HGB, hemoglobin; GWAS, genome-wide association study; ZF1, Zhufeng 1; PCA, principal component analysis; ADMIXTURE, ancestry analysis by ADMIXTURE software; CMS, composite of multiple signal; FST, fixation index; ΔDAF, difference of derived allele frequency; iHS, integrated haplotype score; XP-EHH, cross-population extended haplotype homozygosity.

**Figure 1** illustrates the design and construction of the HLR, which integrates comprehensive data on samples, genomes, genotypes, phenotypes, genome-wide association study (GWAS) results, population genetic structure, and signals of natural selection in indigenous highlanders from the three major high-altitude regions (Table S1). This unified platform enables researchers to systematically explore and analyze high-altitude human population datasets. HLR consists of two core functional modules: information visualization and online analysis (Figure 1), providing both intuitive data presentation and user-friendly tools for customized research applications.

### Information visualization

#### Sample distribution

The “Samples” module presents the geographic distribution of all 23,336 highlander individuals included in this study. These samples are mapped across the world’s three major high-altitude regions: the Qinghai-Tibet Plateau (Asia), the Andean Plateau (South America), and the Ethiopian Plateau (Africa). A global map displays sample locations, with zoomable regions that allow users to explore the finer-scale distribution of subpopulations within each plateau. Detailed information about each sampling site is provided in a table below the map, enabling easy access to population metadata.

#### Phenotypic pattern

The “Traits” module offers an interactive visualization platform for examining phenotypic patterns among highlander populations. This module is structured into three key components. (1) Correlation with altitude: this section displays the relationship between various traits and altitude in both indigenous highlanders (Tibetans) and non-indigenous highlanders (Han Chinese). Curves are fitted using LOESS regression in R and quantified by correlation coefficients (*r*) and *P* values. (2) Cross-population difference: this component highlights phenotypic differences between Tibetans and Han Chinese living at similar altitudes, categorized into five elevation intervals (1–2 km, 2–3 km, 3–4 km, 4–5 km, and 5–6 km). Differences are quantified using effect size (*β*), standard error (SE), and *P* values, with heterogeneity indicated by *Q*-statistics (*Q*) and their significance (*P*). (3) Cross-sex difference: This section displays sex-stratified trait comparisons between Tibetans and Han Chinese at mid altitude (3–4 km).

The platform supports interactive exploration. For example, to view data on hemoglobin (HGB), users can navigate via the “Blood” category or search “HGB” or “hemoglobin”. Three major observations were made for HGB. (1) HGB levels rise with altitude in both populations, increasing from < 150 g/l to > 200 g/l. The increase is steeper in Han Chinese (*r* = 0.709, *P* = 3.2E−4) than in Tibetans (*r* = 0.649, *P* = 6.1E−4). (2) At 3–4 km elevation, Tibetans exhibit significantly lower HGB levels than Han Chinese (*P* = 2.1E−68), while no significant difference is observed at 1–2 km (*P* = 0.21). (3) Cross-population HGB differences are more pronounced in males (*P* = 2.6E−63) than in females (*P* = 3.6E−36). Additionally, HLR reveals novel adaptive phenotypes not previously reported. For instance, Tibetans exhibit a lower prevalence of myopia, as well as reduced central corneal thickness (CCT; *β* = −38.11, *P* = 1.8E−24) and intraocular pressure (IOP; *β* = −0.86, *P* = 9.2E−5), potentially reflecting adaptation to high UV exposure [13,14].

#### Genome-wide association

The “Associations” module provides genome-wide genotype–phenotype association in highlanders through querying variants, traits, or genes. The general query result displays matched GWASs and related study summaries, including trait name, *P* value, associated allele, physical position, mapped gene, and functional consequence of variant, as well as other related traits about this variant. HLR provides GWAS results from three highlander cohorts (Tibetan, Andean, and Ethiopian). Users can retrieve association information for a specific cohort by selecting it from the dropdown menu.

This module facilitates the identification of highlander-specific genetic features by comparison with public GWAS datasets. Among the 2262 genome-wide significant single-nucleotide variants (SNVs; *P* < 5E−8) identified in Tibetans, 843 (37%) are found in the GWAS Catalog (https://www.ebi.ac.uk/gwas/), while 1419 (63%) are novel (Table S2). These novel associations underscore the value of conducting GWAS in underrepresented populations and offer critical insights into local adaptation and population-specific variants not captured by studies focusing on European cohorts.

#### Variants and genes

The “Variants” and “Genes” modules offer comprehensive annotations for genetic variants and genes in highlander populations. Key features include: (1) variant-level data: type, position, functional consequences, allele frequencies across different subpopulations, and natural selection metrics; (2) gene-level data: all associated traits and variants identified through highlander GWASs; and (3) genome browser access: sequence context and flanking regions for each gene or variant based on the highland reference genome.

These modules allow comparisons between highlanders and lowlanders (*e.g.*, Han Chinese), as well as among highland subpopulations, supporting studies on altitude adaptation and convergent evolution.

#### Genetic structure and natural selection signature

The “PopGen” module includes two submodules: “Genetic Structure” and “Natural Selection”.

The “Genetic Structure” presents principal component analysis (PCA) and ADMIXTURE results to visualize genetic relationships among populations. PCA plots highlight the genetic divergence between Tibetans and other East Asian groups. Users can select populations from the legend to view specific group comparisons. ADMIXTURE results show ancestry composition for *K* values ranging from 2 to 10 (default *K* = 5), with interactive plots that allow toggling between *K* values.

The “Natural Selection” displays genome-wide signatures of positive selection in the Tibetan genome using five statistics: fixation index (FST), difference of derived allele frequency (ΔDAF), integrated haplotype score (iHS), cross-population extended haplotype homozygosity (XP-EHH), and composite of multiple signal (CMS). Both Manhattan plots and tables list variants with top selection signals. Users can hover over plot points or search within tables to access detailed variant information.

#### Genome browser

The “Genome” module features a genome browser built on the high-quality Tibetan genome assembly [6]. This browser displays whole-genome alignments with the human reference genome (GRCh37), enabling users to visualize Tibetan-specific genetic features, including: (1) structural variants identified via long-read sequencing; (2) unique variants in the Tibetan genome; and (3) trait-associated variants from Tibetan GWAS results. Interactive elements allow users to click on a gene or variant symbol to open a detailed information panel. The genome browser facilitates exploration of genetic variation and annotations across specific Tibetan genome regions.

### Online analysis

#### Genotype imputation

Genotype imputation is a critical step in genomic analysis, particularly for underrepresented populations lacking adequate reference panels. To address this, our previous study developed a Tibetan-specific reference panel (1KTGP), comprising 1001 high-coverage genome sequences. This panel has demonstrated superior imputation accuracy for Tibetan genotyping array data compared to global references [7].

To enable wider use of this resource, HLR integrates 1KTGP as an online imputation tool. Users can upload genotype data, perform imputation, monitor task progress, and download results directly through the HLR platform. Full documentation and code for implementing the imputation pipeline are available on the “Documents” page. This feature empowers researchers to conduct accurate genotype inference, facilitating genetic and medical studies in high-altitude populations.

#### Knowledge graph

HLR features a dynamic knowledge graph for exploring the complex relationships among variants, genes, and phenotypic traits in highlander populations. The graph consists of three node types: variant loci, phenotypic traits, and associated genes. Edges connecting these nodes are color-coded based on the statistical significance of genotype–phenotype associations. Users can adjust a *P* value threshold to control the density of the network, focusing on the most significant associations.

This interactive graph allows users to select a node of interest to dynamically expand its network and access detailed information on related entities. Clicking on any node redirects users to a dedicated page with comprehensive annotations and association results. This tool enhances data exploration and aids in the discovery of novel, potentially functional associations that may not have been identified through conventional analysis.

## Summary and future directions

Highlander populations represent a biologically distinct group that has undergone extensive physiological and genetic adaptations to the most extreme environments on Earth. Over the past decades, significant progress has been made in understanding these adaptations in the Qinghai-Tibet Plateau, the Andean Plateau, and the Ethiopian Plateau [2–4,15,16]. The increasing availability of large-scale genomic datasets from these regions [7,8,17–19] offers unprecedented opportunities to investigate the molecular underpinnings of high-altitude adaptation.

Despite these advances, there has been a lack of comprehensive platforms dedicated to organizing and analyzing such data. HLR fills this gap by integrating diverse datasets and offering a user-friendly interface to explore phenotypes, genetic structure, and genomic associations. Additionally, it provides powerful analytical tools such as genotype imputation using population-specific references.

Currently, HLR includes data from 23,336 highlander individuals, predominantly from Tibetan populations. This reflects a current imbalance in the availability of genomic and phenomic data across high-altitude regions. To address this, future updates of HLR will incorporate a broader array of populations from other plateaus, including the Andean Plateau, Ethiopian Plateau, and lesser-studied regions such as the Pamir Plateau, Colorado Plateau, and Deccan Plateau. Expansion plans also include the integration of multi-omics datasets — transcriptomics, epigenomics, and proteomics — from highlander tissues and cells, as well as the inclusion of more detailed and standardized phenotypic data. These enhancements aim to provide a more holistic understanding of the biology of high-altitude adaptation.

Ethnic diversity and representation are critical in genomic and biomedical research, especially for isolated, indigenous populations that have historically been underrepresented [20–22]. HLR seeks to address this gap by prioritizing high-altitude populations and implementing ethically conscious, flexible data-sharing policies. Moving forward, HLR will foster global collaboration, ensure transparent governance, and evolve into a comprehensive multi-omics resource for advancing precision medicine and evolutionary genetics. By enabling deeper insights into highland adaptation and health, HLR is poised to make impactful contributions to science and medicine — both for highlander communities and for broader human populations.

## Data collection and processing

### Data collection

The 17 datasets of highland human populations were collected from 15 published studies (Table S1). A total of 23,336 highland individuals are included, covering three major indigenous highland populations residing in the three high-altitude areas, including 21,101 highlanders from the Qinghai-Tibet Plateau, 1857 highlanders from the Andean Plateau, and 378 highlanders from the Ethiopian Plateau. Among these samples, there are 14,884 individuals with phenotype data, 9433 with GWAS data, 4506 with genome-wide genotype data, and a high-quality highlander genome. Detailed information about the data is presented in Figure S1 and Table S1.

For the phenotype data, we collected 14,884 subjects (12,339 Tibetans and 2545 Han Chinese) who lived in the Qinghai-Tibet Plateau [10]. For each sample, a total of 185 traits of 14 organ systems were measured, including heart (29 traits), lung (7 traits), liver (19 traits), blood (28 traits), kidney (5 traits), spleen (2 traits), gallbladder (3 traits), eye (29 traits), body (7 traits), skin (9 traits), lipid (5 traits), thyroid (4 traits), metabolism (23 traits), and reproduction (15 traits).

For the genotype data, we collected two types of data, including WGS genotype data and WGA genotype data. The WGS data were collected from 1001 Tibetan individuals with an average sequencing depth of 11.8× [7], recruited from 83 different geographic locations of the Qinghai-Tibet Plateau. The WGA genotype data included 3008 Tibetan individuals [8], 429 Quechua individuals from the Andean Plateau [18], and 68 individuals (26 Amhara, 21 Oromo, and 21 Tigray) from the Ethiopian Plateau [19]. Additionally, we obtained a high-quality Tibetan reference genome (the ZF1 genome assembly) and 17,900 structural variants (SVs; variants with length ≥ 50 bp) based on long-read sequencing data [6].

For the GWAS data, the summary statistics of 9433 highlanders from the three plateaus were collected from multiple published studies [8,9,23–30], including 7695 Tibetan highlanders, 1428 Andean highlanders, and 310 Ethiopian highlanders (Figure S1; Table S1).

### Phenotype data processing

#### Data quality control

To ensure consistency in data qualities and phenotype definitions across datasets, we performed re-processing for collected phenotype data. Raw phenotype data (*n* = 14,884, including 12,339 Tibetans and 2545 Han Chinese) were filtered to exclude the following individuals: those with mixed ancestry within three generations; smokers; drinkers; those under 18 or over 70 years old; relatives within three generations (questionnaire survey); hepatitis B/C positive individuals; and those with a family history of known genetic diseases. After filtering, 10,084 individuals were retained for downstream analyses, including 8701 Tibetans and 1383 Han Chinese (Figure S1).

#### Standardization

We standardized the trait terms in HLR by mapping them to biological medical ontologies, including National Cancer Institute Thesaurus (NCIT; https://ncithesaurus.nci.nih.gov/), Experimental Factor Ontology (EFO; https://www.ebi.ac.uk/efo/), and The Vertebrate Trait Ontology (VT; https://www.ebi.ac.uk/ols4/ontologies/vt), among others. Each of these biological medical ontologies covers standardized terms from diseases and traits to anatomical structures and physiological processes. To ensure that each feature of our traits can be accurately mapped to the corresponding ontology term, we made manual check after batch matching by ontology ID.

#### Identification of altitude-related traits

Linear regression models were applied to evaluate the correlation between altitude and trait by the *lm* function of R with age, sex, and ethnicity as covariates. Based on the correlations, we determine whether altitude has a significant impact on phenotypic traits, and we record the changing trends and patterns of various phenotypic traits as altitude increases. Correlation coefficient (*r*) and significance (*P* value) are reported in the “Traits” module.

#### Identification of altitude-adaptation traits

Univariate comparisons of the average of each trait between Tibetans and Han Chinese who lived at the same high altitude were performed using the ANCOVA test, implemented via the *aov* function in R, with sex and age as covariates. Five altitude intervals were included: 1–2 km, 2–3 km, 3–4 km, 4–5 km, and 5–6 km. To ensure robust identification of altitude-adaptation traits, we considered both effect size difference and sample size variability, and we also performed heterogeneity tests (Cochran’s *Q* tests) to compare the effect sizes across groups living at different altitudes. A phenotypic trait with a significant difference (*P* < 0.05) between these two groups likely reflects the highland adaptation of highland Tibetans. We also conducted sex-biased analysis by separating males from females in analysis with age as covariates.

### Genotype data processing

#### Data quality control and integration

To ensure consistency in data quality and obtain high-quality genotypes of highlanders, we implemented a two-step quality control (QC) process for each dataset, including sample-level QC and variant-level QC. First, we filtered samples based on the following criteria: (1) missing rate > 3%; (2) heterozygosity rate beyond ±3 standard deviations from the mean; and (3) duplicates or individuals without mixed ancestry within three generations. Then we filtered the variants based on following criteria: (1) singleton variants; (2) variants with missing rate > 3%; and (3) variants with significant deviation from Hardy-Weinberg equilibrium (*P* < 1E−10). We converted all the genotype data into coordinates of the GRCh37 assembly and integrated the data of the same ethnic group. The raw genotype data included 30,829,034 variants in 4506 samples. After QC and integration, we retained 29,878,207 variants in 4506 samples.

#### Statistics and variant annotations

After QC, we calculated the allele frequency of each variant within each ethnic population and within subgroups at the same sampling location. We also conducted functional annotations of variants using Ensembl Variant Effect Predictor (VEP; v110).

#### Population structure analysis

We merged biallelic SNV genotypes of all highlanders with global populations from the 1000 Genomes Project (1KGP; *n* = 2504) [31] and the Human Genome Diversity Project (HGDP; *n* = 828) [32]. After pruning on the merged dataset using PLINK2 [33], we performed PCA using the *smartPCA* program of EIGENSOFT [34]. Additionally, we ran ADMIXTURE (v1.3.0) by randomly selecting 20 samples for each population from highlanders and global individuals. Five iterations were conducted with a random seed for each value of *K* from 2 to 10.

#### Natural selection detection

Five methods were employed to detect the natural selection signals in the genomes of highlanders: two methods based on haplotype information (XP-EHH and iHS), two methods based on allele frequency (FST and ΔDAF), and the CMS method which combines the four aforementioned statistics to assess natural selection signatures for each variant. Detailed methods have been described in our previous study [7].

### GWAS data processing

#### Re-conducting GWAS using a high-quality highlander panel

We re-conducted GWAS for one public dataset both with genotypes and phenotypes, which includes 3008 Tibetan individuals, 287,691 variants (array genotype data), and 91 quantitative traits per individual. In the original study, Yang et al. [8] imputed this Tibetan array data using the 1000 Genome Project reference panel, which introduced ancestry bias in the imputed genotypes. To address potential reference bias and improve genotype accuracy in the original GWAS, we re-analyzed this Tibetan array data. We used a high-quality Tibetan reference panel 1KTGP [7] with 1001 individuals to re-impute the original Tibetan array data (287,149 variants from HumanCoreExome-12 BeadChip) [8] using IMPUTE2 [35]. Subsequently, we performed QC on the re-imputed data following the published protocol [36], resulting in 3,904,687 variants. GWAS was then conducted on these clean data using GEMMA [37]. In these analyses, we controlled for altitude, sex, and age as covariates.

#### Integrating summary statistics from all datasets

Summary statistics from our GWAS re-analysis were integrated with those from other published highlander GWASs (for which only summary statistics were available), resulting in a combined resource that includes 7695 Tibetan highlanders, 1428 Andean highlanders, and 310 Ethiopian highlanders. All GWAS records were harmonized by standardizing them to the same genome coordinate system (GRCh37), ensuring consistent allele encoding and aligning effect directions. The GWAS result for each genotype–phenotype pair was defined as an association record, characterized by the variant rs-ID, physical position, effect allele, cohort resource, *P* value, associated trait, associated gene, and variant consequence. Associated genes were determined based on their physical proximity to the top associated variants in GWAS results. Variants passing the threshold of *P* < 0.001 are shown in this module, allowing users to find nominally significant associations. Associated genes and traits were consolidated into de-duplicated records and linked to their corresponding association records. This comprehensive database enables researchers to easily access GWAS results by searching for any gene name or trait name. A knowledge graph was constructed to visually represent the relationships between different entities within the database. Furthermore, association records were organized into projects based on the cohorts they originated from, allowing users to efficiently access GWAS results specific to a particular cohort.

### Construction of a genome browser for highlanders

We constructed a genome browser for highland populations based on the high-quality Tibetan assembly ZF1 [6] using JBrowse 2 [38]. Seven tracks were included for a genome region, including the reference sequence based on GRCh37, sequence alignment between ZF1 and GRCh37, SNVs and SVs in the Tibetan genome, gene annotations based on GenCodeR43, clinically/phenotypically associated SNVs based on the published GWAS and highlander GWAS analyses.

### Construction of a highlander-specific imputation reference panel

Utilizing the large-scale WGS data of Tibetan populations [7], we constructed a highland population-specific reference panel for genotype imputation in HLR. The panel comprised 1001 unrelated Tibetan individuals, with a total of 28.2 million autosomal biallelic variants after excluding variants with a genotype missing rate > 5% and singletons/doubletons.SHAPEIT4 (v4.2.2; with default settings) were employed to conduct population-based haplotype phasing. The phased haplotypes of Tibetans were uploaded to HLR as the reference panel.

## Supplementary Material

qzaf083_Supplementary_Data

## Data Availability

HLR is available online for free at https://ngdc.cncb.ac.cn/hiland/. All datasets used in HLR are freely accessible through the “Download” module of HLR. HLR has been submitted to Database Commons [39] at the National Genomics Data Center (NGDC), China National Center for Bioinformation (CNCB), which is publicly accessible at https://ngdc.cncb.ac.cn/databasecommons/database/id/10238.

## CRediT author statement

**Weijie Zhang:** Methodology, Software, Investigation, Data curation, Writing – original draft. **Xiaoning Chen:** Methodology, Software, Investigation, Data curation, Writing – original draft. **Yanling Sun:** Methodology, Software, Writing – review & editing. **Yibo Wang:** Methodology, Software, Investigation, Data curation, Writing – review & editing. **Bixia Tang:** Methodology. **Yu Zhang:** Software. **Kai Liu:** Data curation. **Wemmin Zhao:** Conceptualization, Supervision, Funding acquisition. **Bing Su:** Conceptualization, Supervision, Funding acquisition, Writing – review & editing. **Yaoxi He:** Conceptualization, Writing – original draft, Writing – review & editing, Supervision, Project administration, Funding acquisition. All authors have read and approved the final manuscript.
