# Supplementary material for: HiLand Resource: A Comprehensive Database of Highland Human Populations
Source: Genomics Proteomics Bioinformatics. 2025 Sep 14;23(5):qzaf083. doi: 10.1093/gpbjnl/qzaf083 (PMC12854720; doi:10.1093/gpbjnl/qzaf083)
Supplement: qzaf083_Supplementary_Data [file qzaf083_supplementary_data.zip › Supplementary material captions.docx]

**Supplementary materials**

**Figure S1 Schematic diagram of data composition and processing in HLR database**

**A.** Phenome data information. Two public datasets composite the phenotype data of HLR database, the raw data including 14,884 individuals (12,339 Tibetans and 2545 Han Chinese) living at multiple altitude locations (ranging from 1120 m to 5020 m). After QCs, 10,084 samples were left for downstream analysis, including 8701 Tibetans and 1383 Han Chinese. **B.** Genome data information. Five public datasets were included in the HLR, compassing the 4507 highlanders and 30,829,034 variants for raw data. After sample QCs and variant QCs, 4507 samples and 29,878,206 variants were retained to clean data for HLR and downstream genetic analysis. **C.** GWAS data information. Ten public datasets were involved in HLR. We collected raw data (genotypes and phenotypes) of Dataset-09, then re-conducted the GWAS by imputing genotypes using 1KTGP, a Tibetan-specific panel established at HLR. The remaining nine datasets are GWAS summary statistics. **D.** Modules of HLR. **E.** Contents of each module in HLR. GWAS, genome-wide association study; QC, quality control; WGS, whole-genome sequencing; HWE, Hardy-Weinberg equilibrium; SD, standard deviation.

**Table S1 Data summary of HLR database**

**Table S2 1419 newly identified associations in HLR**
