# Supplementary material for: HiLand Resource: A Comprehensive Database of Highland Human Populations
Source: Genomics Proteomics Bioinformatics. 2025 Sep 14;23(5):qzaf083. doi: 10.1093/gpbjnl/qzaf083 (PMC12854720; doi:10.1093/gpbjnl/qzaf083)
Supplement: qzaf083_Supplementary_Data [file qzaf083_supplementary_data.zip › Table S1.docx]

**Table S1 Data summary of HLR database**

| **Project ID** | **Highlands** | **Data types** | **Project name** | **Datasets** | **Sample size** | | **Populations** | **Ref.** | **Genotype** | **Phenotype** | **Genome** | **GWAS** |
| --- | --- | --- | --- | --- | --- | --- | --- | --- | --- | --- | --- | --- |
| Project-01 | Qinghai-Tibet Plateau | Phenome data | Phenome of 12,404 highland Tibetan and Han | Dataset-01 | 12,404 | 21,101 | Tibetan/Han Chinese | <https://doi.org/10.1016/j.isci.2023.107677> | - | 12,404 | - | - |
| Project-02 | Qinghai-Tibet Plateau | Genome data | A high-quality genome of Tibetans | Dataset-08 | 1 |  | Tibetan | <https://doi.org/10.1093/nsr/nwz160> | - | - | 1 | - |
| Project-03 | Qinghai-Tibet Plateau | WGS data | WGS of 1001 Tibetans | Dataset-04 | 1001 |  | Tibetan | <https://doi.org/10.1186/s13059-023-02912-1> | 4009 | - | - | - |
| Project-04 | Qinghai-Tibet Plateau | WGA data | WGA data of 3008 Tibetans | Dataset-05 | 3008 |  | Tibetan | <https://doi.org/10.1073/pnas.1617042114> |  | - | - | - |
| Project-04 | Qinghai-Tibet Plateau | GWAS data | GWAS data of 3008 Tibetans | Dataset-09 |  |  | Tibetan | <https://doi.org/10.1073/pnas.1617042114> | - | - | - | 7695 |
| Project-05 | Qinghai-Tibet Plateau | GWAS data | GWAS data of 2182 pregnant Tibetans | Dataset-10 | 2480 |  | Tibetan | <https://doi.org/10.1016/j.cub.2023.08.021> | - | - | - |  |
| Project-05 | Qinghai-Tibet Plateau | Phenome data | Phenotypes of 2480 highland Tibetan and Han | Dataset-02 |  |  | Tibetan/Han Chinese | <https://doi.org/10.1016/j.cub.2023.08.021> | - | 2480 | - |  |
| Project-07 | Qinghai-Tibet Plateau | GWAS data | GWAS data of 1000 Tibetan women | Dataset-11 | 1000 |  | Tibetan | <https://doi.org/10.1371/journal.pgen.1007650> | - | - | - |  |
| Project-08 | Qinghai-Tibet Plateau | GWAS data | GWAS data of 288 Tibetans | Dataset-12 | 288 |  | Tibetan | <https://doi.org/10.1073/pnas.1422759112> | - | - | - |  |
| Project-09 | Qinghai-Tibet Plateau | GWAS data | GWAS data of 203 Tibetans | Dataset-13 | 203 |  | Tibetan | <https://doi.org/10.1089/ham.2015.0065> | - | - | - |  |
| Project-10 | Qinghai-Tibet Plateau | GWAS data | GWAS data of 400 Tibetans | Dataset-14 | 400 |  | Tibetan | <https://doi.org/10.1080/07391102.2019.1711190> | - | - | - |  |
| Project-11 | Qinghai-Tibet Plateau | GWAS data | GWAS data of 316 Tibetans | Dataset-15 | 316 |  | Tibetan | <https://doi.org/10.1371/journal.pone.0269671> | - | - | - |  |
| Project-12 | Andean Plateau | WGA data | WGA data of 429 Quechua | Dataset-06 | 429 |  | Andean | <https://doi.org/10.1073/pnas.1906171116> | 429 | - | - |  |
| Project-13 | Andean Plateau | GWAS data | GWAS data of 421 Andeans | Dataset-16 | 421 | 1857 | Andean | <https://doi.org/10.1016/j.ajhg.2022.04.014> | - | - | - | - |
| Project-13 | Andean Plateau | GWAS data | GWAS data of 86 Andeans | Dataset-16  Dataset-17 | 86 |  | Andean | <https://doi.org/10.1016/j.ajhg.2022.04.014> | - | - | - | 1428 |
| Project-13 | Andean Plateau | GWAS data | GWAS data of 324 Andeans |  | 324 |  | Andean | <https://doi.org/10.1016/j.ajhg.2022.04.014> | - | - | - |  |
| Project-13 | Andean Plateau | GWAS data | GWAS data of 285 Andeans |  | 285 |  | Andean | <https://doi.org/10.1016/j.ajhg.2022.04.014> | - | - | - |  |
| Project-14 | Andean Plateau | GWAS data | GWAS data of 312 Andeans |  | 312 |  | Andean | <https://doi.org/10.3389/fgene.2019.00690> | - | - | - |  |
| Project-15 | Ethiopian Plateau | WGA data | WGA data of 26 Ethiopian Amhara | Dataset-07 | 26 |  | Ethiopian | <https://doi.org/10.1016/j.ajhg.2012.05.015> | 68 | - | - |  |
| Project-15 | Ethiopian Plateau | WGA data | WGA data of 21 Ethiopian Tigray | Dataset-07  Dataset-18 | 21 | 378 | Ethiopian | <https://doi.org/10.1016/j.ajhg.2012.05.015> | 68  - | - | - | - |
| Project-15 | Ethiopian Plateau | WGA data | WGA data of 21 Ethiopian Oromo |  | 21 |  | Ethiopian | <https://doi.org/10.1016/j.ajhg.2012.05.015> |  | - | - | - |
| Project-16 | Ethiopian Plateau | GWAS data | GWAS data of 192 Ethiopian Amhara |  | 192 |  | Ethiopian | <https://doi.org/10.1371/journal.pgen.1003110> |  | - | - | - |
| Project-16 | Ethiopian Plateau | GWAS data | GWAS data of 118 Ethiopian Oromo | Dataset-18 | 118 |  | Ethiopian | <https://doi.org/10.1371/journal.pgen.1003110> | - | - | - | 310 |
| Total |  |  |  |  | 23,336 |  |  |  | 4506 | 14,884 | 1 | 9433 |
