# Supplementary material for: HiLand Resource: A Comprehensive Database of Highland Human Populations
Source: Genomics Proteomics Bioinformatics. 2025 Sep 14;23(5):qzaf083. doi: 10.1093/gpbjnl/qzaf083 (PMC12854720; doi:10.1093/gpbjnl/qzaf083)
Supplement: qzaf083_Supplementary_Data [file qzaf083_supplementary_data.zip › Table S2.docx]

**Table S2 1419 newly identified associations in HLR**

| **Chr** | **Position (GRCh37)** | **Associated trait** | | **Effect size** | ***P* value** |
| --- | --- | --- | --- | --- | --- |
| 6 | 160994326 | | Lipoprotein | 281.16 | 1.40E-45 |
| 2 | 234586814 | | Total bilirubin | 1.95 | 7.02E-41 |
| 2 | 234603439 | | Total bilirubin | 1.95 | 2.96E-40 |
| 2 | 234593374 | | Total bilirubin | 1.93 | 3.65E-40 |
| 2 | 234583062 | | Total bilirubin | 1.94 | 4.51E-40 |
| 2 | 234587442 | | Total bilirubin | 1.95 | 7.70E-40 |
| 2 | 234602906 | | Total bilirubin | 1.94 | 9.76E-40 |
| 2 | 234583183 | | Total bilirubin | 1.92 | 2.08E-39 |
| 2 | 234597915 | | Total bilirubin | 1.90 | 4.50E-39 |
| 2 | 234589504 | | Total bilirubin | 1.91 | 4.86E-39 |
| 2 | 234588045 | | Total bilirubin | 1.91 | 1.63E-38 |
| 2 | 234584306 | | Total bilirubin | 1.91 | 2.59E-38 |
| 2 | 234588585 | | Total bilirubin | 1.88 | 9.56E-38 |
| 2 | 234586814 | | Indirect bilirubin | 1.23 | 9.88E-38 |
| 2 | 234603439 | | Indirect bilirubin | 1.24 | 1.08E-37 |
| 2 | 234593374 | | Indirect bilirubin | 1.22 | 2.08E-37 |
| 2 | 234590970 | | Total bilirubin | 1.86 | 3.17E-37 |
| 2 | 234590974 | | Total bilirubin | 1.86 | 3.17E-37 |
| 2 | 234590975 | | Total bilirubin | 1.86 | 3.17E-37 |
| 2 | 234583062 | | Indirect bilirubin | 1.23 | 3.54E-37 |
| 2 | 234587442 | | Indirect bilirubin | 1.23 | 9.81E-37 |
| 2 | 234602906 | | Indirect bilirubin | 1.22 | 1.21E-36 |
| 2 | 234588045 | | Indirect bilirubin | 1.22 | 1.79E-36 |
| 2 | 234583183 | | Indirect bilirubin | 1.21 | 2.13E-36 |
| 2 | 234589504 | | Indirect bilirubin | 1.21 | 2.35E-36 |
| 2 | 234597915 | | Indirect bilirubin | 1.19 | 5.86E-36 |
| 2 | 234592635 | | Total bilirubin | 1.84 | 6.61E-36 |
| 2 | 234586814 | | Direct bilirubin | 0.67 | 7.66E-36 |
| 2 | 234584306 | | Indirect bilirubin | 1.21 | 8.01E-36 |
| 2 | 234587442 | | Direct bilirubin | 0.68 | 1.66E-35 |
| 2 | 234590974 | | Indirect bilirubin | -0.99 | 2.36E-35 |
| 2 | 234590975 | | Indirect bilirubin | -0.99 | 2.36E-35 |
| 2 | 234602906 | | Indirect bilirubin | -0.99 | 2.40E-35 |
| 2 | 234602906 | | Direct bilirubin | 0.67 | 3.46E-35 |
| 2 | 234590970 | | Indirect bilirubin | -0.99 | 3.50E-35 |
| 2 | 234583062 | | Direct bilirubin | 0.67 | 4.78E-35 |
| 2 | 234588045 | | Direct bilirubin | 0.67 | 5.26E-35 |
| 2 | 234588585 | | Indirect bilirubin | 1.19 | 5.78E-35 |
| 6 | 160913727 | | Lipoprotein | 196.48 | 6.16E-35 |
| 2 | 234589504 | | Direct bilirubin | 0.66 | 6.17E-35 |
| 2 | 234583183 | | Direct bilirubin | 0.67 | 6.59E-35 |
| 2 | 234603439 | | Direct bilirubin | 0.67 | 6.85E-35 |
| 2 | 234593374 | | Direct bilirubin | 0.66 | 7.33E-35 |
| 2 | 234587442 | | Indirect bilirubin | -0.98 | 8.04E-35 |
| 2 | 234597915 | | Direct bilirubin | 0.66 | 8.72E-35 |
| 2 | 234586974 | | Indirect bilirubin | -0.98 | 1.16E-34 |
| 2 | 234584306 | | Indirect bilirubin | -0.98 | 1.54E-34 |
| 2 | 234588585 | | Indirect bilirubin | -0.98 | 1.84E-34 |
| 2 | 234593374 | | Indirect bilirubin | -0.98 | 2.11E-34 |
| 2 | 234589504 | | Indirect bilirubin | -0.98 | 2.19E-34 |
| 6 | 160970833 | | Lipoprotein | 194.61 | 3.19E-34 |
| 2 | 234590970 | | Indirect bilirubin | 1.17 | 3.62E-34 |
| 2 | 234590974 | | Indirect bilirubin | 1.17 | 3.62E-34 |
| 2 | 234590975 | | Indirect bilirubin | 1.17 | 3.62E-34 |
| 2 | 234592635 | | Indirect bilirubin | -0.97 | 4.65E-34 |
| 2 | 234586981 | | Indirect bilirubin | 0.98 | 5.77E-34 |
| 2 | 234588045 | | Indirect bilirubin | -0.97 | 6.65E-34 |
| 2 | 234586983 | | Indirect bilirubin | -0.97 | 8.43E-34 |
| 2 | 234583183 | | Indirect bilirubin | -0.97 | 9.97E-34 |
| 2 | 234597915 | | Indirect bilirubin | -0.97 | 1.32E-33 |
| 2 | 234586814 | | Indirect bilirubin | -0.96 | 2.13E-33 |
| 2 | 234603439 | | Indirect bilirubin | -0.96 | 2.25E-33 |
| 2 | 234588585 | | Direct bilirubin | 0.65 | 2.81E-33 |
| 2 | 234592635 | | Indirect bilirubin | 1.16 | 3.34E-33 |
| 2 | 234581834 | | Indirect bilirubin | -0.95 | 3.96E-33 |
| 2 | 234584306 | | Direct bilirubin | 0.65 | 4.07E-33 |
| 2 | 234583062 | | Indirect bilirubin | -0.96 | 4.48E-33 |
| 2 | 234590974 | | Total bilirubin | -1.41 | 9.32E-33 |
| 2 | 234590975 | | Total bilirubin | -1.41 | 9.32E-33 |
| 2 | 234590970 | | Direct bilirubin | 0.64 | 9.60E-33 |
| 2 | 234590974 | | Direct bilirubin | 0.64 | 9.60E-33 |
| 2 | 234590975 | | Direct bilirubin | 0.64 | 9.60E-33 |
| 2 | 234590970 | | Total bilirubin | -1.40 | 1.19E-32 |
| 2 | 234602906 | | Total bilirubin | -1.40 | 2.17E-32 |
| 2 | 234587442 | | Total bilirubin | -1.40 | 2.46E-32 |
| 2 | 234592635 | | Direct bilirubin | 0.64 | 4.67E-32 |
| 2 | 234582504 | | Indirect bilirubin | -0.93 | 5.71E-32 |
| 2 | 234586974 | | Total bilirubin | -1.39 | 5.74E-32 |
| 2 | 234589504 | | Total bilirubin | -1.39 | 6.18E-32 |
| 2 | 234592635 | | Total bilirubin | -1.39 | 7.09E-32 |
| 2 | 234597915 | | Total bilirubin | -1.40 | 7.30E-32 |
| 2 | 234588585 | | Total bilirubin | -1.39 | 8.04E-32 |
| 2 | 234593374 | | Total bilirubin | -1.39 | 8.98E-32 |
| 2 | 234586981 | | Total bilirubin | 1.39 | 1.36E-31 |
| 2 | 234588045 | | Total bilirubin | -1.39 | 1.41E-31 |
| 2 | 234584306 | | Total bilirubin | -1.38 | 1.67E-31 |
| 2 | 234581748 | | Indirect bilirubin | -0.92 | 1.71E-31 |
| 2 | 234581654 | | Indirect bilirubin | -0.92 | 2.12E-31 |
| 2 | 234583183 | | Total bilirubin | -1.38 | 2.23E-31 |
| 2 | 234586983 | | Total bilirubin | -1.38 | 2.39E-31 |
| 2 | 234603439 | | Total bilirubin | -1.37 | 6.43E-31 |
| 2 | 234586814 | | Total bilirubin | -1.37 | 6.69E-31 |
| 2 | 234581834 | | Total bilirubin | -1.35 | 8.04E-31 |
| 2 | 234583062 | | Total bilirubin | -1.36 | 1.62E-30 |
| 2 | 234581748 | | Total bilirubin | -1.34 | 3.55E-30 |
| 2 | 234581654 | | Total bilirubin | -1.33 | 4.34E-30 |
| 2 | 234591987 | | Indirect bilirubin | -0.90 | 5.48E-30 |
| 2 | 234582504 | | Total bilirubin | -1.32 | 7.57E-30 |
| 6 | 160788568 | | Lipoprotein | 174.75 | 5.42E-29 |
| 6 | 160727824 | | Lipoprotein | 177.77 | 4.46E-28 |
| 2 | 234591987 | | Total bilirubin | -1.28 | 5.22E-28 |
| 6 | 160711017 | | Lipoprotein | 170.75 | 4.13E-27 |
| 2 | 234590970 | | Direct bilirubin | -0.47 | 5.22E-25 |
| 2 | 234590974 | | Direct bilirubin | -0.47 | 5.56E-25 |
| 2 | 234590975 | | Direct bilirubin | -0.47 | 5.56E-25 |
| 2 | 234602906 | | Direct bilirubin | -0.48 | 5.80E-25 |
| 2 | 234603439 | | Direct bilirubin | -0.48 | 6.12E-25 |
| 2 | 234584306 | | Direct bilirubin | -0.48 | 6.26E-25 |
| 2 | 234592635 | | Direct bilirubin | -0.47 | 7.13E-25 |
| 2 | 234587442 | | Direct bilirubin | -0.47 | 7.38E-25 |
| 2 | 234589504 | | Direct bilirubin | -0.47 | 1.21E-24 |
| 2 | 234593374 | | DBIL/TBIL ratio | -0.02 | 1.51E-24 |
| 2 | 234597915 | | Direct bilirubin | -0.47 | 1.66E-24 |
| 2 | 234588045 | | Direct bilirubin | -0.47 | 1.72E-24 |
| 2 | 234588585 | | Direct bilirubin | -0.47 | 3.45E-24 |
| 2 | 234593374 | | Direct bilirubin | -0.47 | 3.82E-24 |
| 2 | 234602641 | | Total bilirubin | 1.66 | 5.19E-24 |
| 2 | 234582504 | | Direct bilirubin | -0.46 | 5.59E-24 |
| 2 | 234583846 | | Total bilirubin | 1.66 | 5.69E-24 |
| 2 | 234602906 | | DBIL/TBIL ratio | -0.02 | 7.15E-24 |
| 2 | 234584306 | | DBIL/TBIL ratio | -0.02 | 7.43E-24 |
| 2 | 234583062 | | DBIL/TBIL ratio | -0.02 | 9.25E-24 |
| 2 | 234586814 | | Direct bilirubin | -0.46 | 1.03E-23 |
| 2 | 234581834 | | Direct bilirubin | -0.46 | 1.13E-23 |
| 2 | 234583183 | | Direct bilirubin | -0.46 | 2.02E-23 |
| 2 | 234583062 | | Direct bilirubin | -0.46 | 2.25E-23 |
| 2 | 234589504 | | DBIL/TBIL ratio | -0.02 | 3.87E-23 |
| 2 | 234587442 | | DBIL/TBIL ratio | -0.02 | 4.50E-23 |
| 2 | 234588045 | | DBIL/TBIL ratio | -0.02 | 4.51E-23 |
| 2 | 234586981 | | Direct bilirubin | 0.46 | 4.84E-23 |
| 2 | 234581748 | | Direct bilirubin | -0.45 | 5.88E-23 |
| 2 | 234586814 | | DBIL/TBIL ratio | -0.02 | 6.11E-23 |
| 2 | 234586974 | | Direct bilirubin | -0.45 | 7.16E-23 |
| 2 | 234603439 | | DBIL/TBIL ratio | -0.02 | 7.67E-23 |
| 2 | 234578762 | | Indirect bilirubin | -0.81 | 7.91E-23 |
| 2 | 234586983 | | Direct bilirubin | -0.45 | 8.85E-23 |
| 2 | 234581654 | | Direct bilirubin | -0.45 | 8.99E-23 |
| 2 | 234602641 | | Indirect bilirubin | 1.06 | 9.70E-23 |
| 2 | 234579892 | | Total bilirubin | 1.59 | 1.31E-22 |
| 2 | 234597915 | | DBIL/TBIL ratio | -0.02 | 1.48E-22 |
| 2 | 234602641 | | Direct bilirubin | 0.59 | 1.62E-22 |
| 2 | 234583846 | | Indirect bilirubin | 1.05 | 1.63E-22 |
| 2 | 234588585 | | DBIL/TBIL ratio | -0.02 | 2.38E-22 |
| 2 | 234583183 | | DBIL/TBIL ratio | -0.02 | 6.39E-22 |
| 2 | 234517782 | | Indirect bilirubin | -0.80 | 1.01E-21 |
| 2 | 234579892 | | Indirect bilirubin | 1.02 | 1.36E-21 |
| 2 | 234592635 | | DBIL/TBIL ratio | -0.02 | 1.50E-21 |
| 2 | 234583846 | | Direct bilirubin | 0.58 | 1.64E-21 |
| 2 | 234552292 | | Indirect bilirubin | -0.79 | 2.19E-21 |
| 2 | 234525355 | | Indirect bilirubin | -0.79 | 2.46E-21 |
| 2 | 234578762 | | Total bilirubin | -1.15 | 3.67E-21 |
| 2 | 234583490 | | Total bilirubin | 1.54 | 3.94E-21 |
| 2 | 234535007 | | Indirect bilirubin | -0.78 | 3.97E-21 |
| 2 | 234556326 | | Indirect bilirubin | -0.78 | 4.70E-21 |
| 2 | 234579892 | | Direct bilirubin | 0.57 | 4.74E-21 |
| 2 | 234552292 | | Total bilirubin | -1.16 | 5.86E-21 |
| 2 | 234590970 | | DBIL/TBIL ratio | -0.02 | 5.99E-21 |
| 2 | 234590974 | | DBIL/TBIL ratio | -0.02 | 5.99E-21 |
| 2 | 234590975 | | DBIL/TBIL ratio | -0.02 | 5.99E-21 |
| 2 | 234525355 | | Total bilirubin | -1.16 | 7.19E-21 |
| 2 | 234539381 | | Indirect bilirubin | -0.78 | 8.14E-21 |
| 2 | 234556326 | | Total bilirubin | -1.15 | 8.57E-21 |
| 2 | 234535007 | | Total bilirubin | -1.15 | 9.79E-21 |
| 2 | 234527397 | | Indirect bilirubin | -0.78 | 1.03E-20 |
| 6 | 160753038 | | Lipoprotein | 106.26 | 1.45E-20 |
| 2 | 234534869 | | Total bilirubin | -1.15 | 1.57E-20 |
| 2 | 234591987 | | Direct bilirubin | -0.42 | 1.68E-20 |
| 2 | 234643560 | | Indirect bilirubin | 1.93 | 1.77E-20 |
| 2 | 234517782 | | Total bilirubin | -1.15 | 2.06E-20 |
| 2 | 234527397 | | Total bilirubin | -1.14 | 3.55E-20 |
| 2 | 234539381 | | Total bilirubin | -1.14 | 3.65E-20 |
| 2 | 234512976 | | Indirect bilirubin | -0.78 | 3.85E-20 |
| 2 | 234534869 | | Indirect bilirubin | -0.76 | 4.03E-20 |
| 2 | 234548334 | | Indirect bilirubin | -0.77 | 4.60E-20 |
| 2 | 234583490 | | Indirect bilirubin | 0.98 | 5.18E-20 |
| 2 | 234572783 | | Total bilirubin | -1.12 | 5.40E-20 |
| 2 | 234643560 | | Total bilirubin | 2.90 | 6.83E-20 |
| 2 | 234550970 | | Indirect bilirubin | -0.76 | 8.44E-20 |
| 6 | 160744406 | | Lipoprotein | 104.52 | 9.24E-20 |
| 2 | 234548334 | | Total bilirubin | -1.13 | 1.02E-19 |
| 2 | 234512976 | | Total bilirubin | -1.14 | 1.07E-19 |
| 2 | 234572783 | | Indirect bilirubin | -0.75 | 1.14E-19 |
| 2 | 234513720 | | Indirect bilirubin | -0.76 | 1.28E-19 |
| 2 | 234513720 | | Total bilirubin | -1.13 | 1.37E-19 |
| 2 | 234583490 | | Direct bilirubin | 0.55 | 1.38E-19 |
| 6 | 160748088 | | Lipoprotein | 103.14 | 1.43E-19 |
| 2 | 234534648 | | Indirect bilirubin | -0.75 | 2.36E-19 |
| 2 | 234537447 | | Indirect bilirubin | -0.75 | 2.59E-19 |
| 2 | 234537447 | | Total bilirubin | -1.12 | 2.71E-19 |
| 2 | 234648860 | | Indirect bilirubin | 1.84 | 2.86E-19 |
| 2 | 234550970 | | Total bilirubin | -1.11 | 2.86E-19 |
| 2 | 234534648 | | Total bilirubin | -1.10 | 3.07E-19 |
| 2 | 234565438 | | Indirect bilirubin | -0.74 | 3.68E-19 |
| 2 | 234512005 | | Indirect bilirubin | -0.75 | 3.73E-19 |
| 2 | 234565438 | | Total bilirubin | -1.10 | 5.70E-19 |
| 2 | 234583846 | | Indirect bilirubin | 0.78 | 5.77E-19 |
| 2 | 234529391 | | Indirect bilirubin | -0.75 | 6.05E-19 |
| 2 | 234648860 | | Total bilirubin | 2.76 | 1.02E-18 |
| 2 | 234512005 | | Total bilirubin | -1.10 | 1.70E-18 |
| 2 | 234529391 | | Total bilirubin | -1.09 | 2.32E-18 |
| 2 | 234638249 | | Total bilirubin | 1.60 | 2.75E-18 |
| 2 | 234583490 | | Indirect bilirubin | 0.77 | 3.05E-18 |
| 2 | 234647478 | | Total bilirubin | 1.62 | 4.08E-18 |
| 2 | 234641908 | | Total bilirubin | 1.61 | 5.20E-18 |
| 2 | 234638690 | | Total bilirubin | 1.65 | 6.54E-18 |
| 2 | 234653184 | | Total bilirubin | 1.61 | 1.13E-17 |
| 2 | 234653192 | | Total bilirubin | 1.61 | 1.13E-17 |
| 2 | 234650193 | | Total bilirubin | 1.56 | 1.35E-17 |
| 2 | 234602641 | | Indirect bilirubin | 0.75 | 1.35E-17 |
| 2 | 234638249 | | Indirect bilirubin | 1.02 | 1.66E-17 |
| 2 | 234635467 | | Total bilirubin | 1.55 | 1.66E-17 |
| 2 | 234665659 | | Total bilirubin | 1.60 | 2.06E-17 |
| 2 | 234579892 | | Indirect bilirubin | 0.75 | 2.18E-17 |
| 2 | 234647478 | | Indirect bilirubin | 1.04 | 2.37E-17 |
| 2 | 234637707 | | Total bilirubin | 1.55 | 3.59E-17 |
| 2 | 234581920 | | Indirect bilirubin | 0.73 | 3.94E-17 |
| 2 | 234641908 | | Indirect bilirubin | 1.02 | 4.07E-17 |
| 2 | 234637192 | | Total bilirubin | 1.56 | 4.27E-17 |
| 2 | 234653192 | | Direct bilirubin | 0.45 | 4.98E-17 |
| 2 | 234504098 | | Indirect bilirubin | -0.68 | 5.00E-17 |
| 2 | 234622429 | | Direct bilirubin | 0.45 | 5.08E-17 |
| 2 | 234665659 | | Indirect bilirubin | 1.04 | 6.30E-17 |
| 2 | 234653184 | | Indirect bilirubin | 1.03 | 6.73E-17 |
| 2 | 234653192 | | Indirect bilirubin | 1.03 | 6.73E-17 |
| 2 | 234655303 | | Direct bilirubin | 0.45 | 6.97E-17 |
| 2 | 234637192 | | Indirect bilirubin | 1.02 | 7.20E-17 |
| 2 | 234650193 | | Indirect bilirubin | 1.00 | 7.35E-17 |
| 2 | 234637803 | | Total bilirubin | 1.56 | 7.66E-17 |
| 2 | 234665659 | | Direct bilirubin | 0.45 | 8.04E-17 |
| 2 | 234620272 | | Total bilirubin | 1.53 | 8.70E-17 |
| 2 | 234644421 | | Total bilirubin | 1.52 | 9.90E-17 |
| 2 | 234653184 | | Direct bilirubin | 0.44 | 1.01E-16 |
| 2 | 234635467 | | Indirect bilirubin | 0.99 | 1.09E-16 |
| 2 | 234622429 | | Total bilirubin | 1.58 | 1.10E-16 |
| 2 | 234650193 | | Direct bilirubin | 0.44 | 1.18E-16 |
| 6 | 161068320 | | Lipoprotein | 99.18 | 1.21E-16 |
| 2 | 234638690 | | Indirect bilirubin | 1.04 | 1.34E-16 |
| 2 | 234637569 | | Total bilirubin | 1.53 | 1.40E-16 |
| 2 | 234549226 | | Indirect bilirubin | 0.72 | 1.86E-16 |
| 2 | 234637707 | | Indirect bilirubin | 0.99 | 2.18E-16 |
| 2 | 234578762 | | Total bilirubin | -1.18 | 2.20E-16 |
| 2 | 234673462 | | Direct bilirubin | 0.44 | 2.50E-16 |
| 2 | 234637853 | | Direct bilirubin | 0.44 | 2.57E-16 |
| 2 | 234539381 | | Direct bilirubin | -0.39 | 2.83E-16 |
| 2 | 234552292 | | Direct bilirubin | -0.39 | 2.94E-16 |
| 2 | 234622429 | | Total bilirubin | 1.12 | 3.07E-16 |
| 2 | 234525355 | | Direct bilirubin | -0.39 | 3.25E-16 |
| 2 | 234620272 | | Direct bilirubin | 0.43 | 3.25E-16 |
| 2 | 234617236 | | Direct bilirubin | 0.43 | 3.31E-16 |
| 2 | 234535007 | | Direct bilirubin | -0.39 | 3.60E-16 |
| 2 | 234673462 | | Total bilirubin | 1.55 | 4.12E-16 |
| 2 | 234637569 | | Indirect bilirubin | 0.99 | 4.24E-16 |
| 1 | 11955587 | | Phosphorus | 2.77 | 4.64E-16 |
| 2 | 234630443 | | Total bilirubin | 1.53 | 4.84E-16 |
| 2 | 234504098 | | Total bilirubin | -0.97 | 4.87E-16 |
| 2 | 234527397 | | Direct bilirubin | -0.39 | 4.97E-16 |
| 2 | 234610912 | | Direct bilirubin | 0.43 | 5.30E-16 |
| 2 | 234634916 | | Direct bilirubin | 0.43 | 5.33E-16 |
| 2 | 234658250 | | Total bilirubin | 1.54 | 5.38E-16 |
| 2 | 234655303 | | Total bilirubin | 1.12 | 5.61E-16 |
| 2 | 234622429 | | Indirect bilirubin | 1.01 | 6.11E-16 |
| 2 | 234513720 | | Direct bilirubin | -0.39 | 6.60E-16 |
| 2 | 234653192 | | Total bilirubin | 1.11 | 6.61E-16 |
| 2 | 234556326 | | Direct bilirubin | -0.39 | 6.72E-16 |
| 2 | 234635467 | | Direct bilirubin | 0.43 | 6.84E-16 |
| 2 | 234643749 | | Total bilirubin | 1.54 | 7.09E-16 |
| 2 | 234637803 | | Indirect bilirubin | 0.99 | 7.21E-16 |
| 2 | 234628529 | | Direct bilirubin | 0.43 | 7.30E-16 |
| 2 | 234617236 | | Total bilirubin | 1.49 | 8.20E-16 |
| 2 | 234643749 | | Indirect bilirubin | 1.01 | 8.23E-16 |
| 2 | 234665659 | | Total bilirubin | 1.11 | 8.25E-16 |
| 2 | 234647478 | | Direct bilirubin | 0.43 | 8.52E-16 |
| 2 | 234673462 | | Indirect bilirubin | 1.00 | 8.64E-16 |
| 2 | 234653184 | | Total bilirubin | 1.11 | 8.88E-16 |
| 2 | 234610539 | | Direct bilirubin | 0.43 | 8.92E-16 |
| 2 | 234620272 | | Indirect bilirubin | 0.97 | 9.12E-16 |
| 2 | 234644421 | | Indirect bilirubin | 0.97 | 1.01E-15 |
| 2 | 234650604 | | Total bilirubin | 1.48 | 1.08E-15 |
| 2 | 234534869 | | Direct bilirubin | -0.38 | 1.12E-15 |
| 2 | 234517782 | | Direct bilirubin | -0.39 | 1.45E-15 |
| 2 | 234637707 | | Direct bilirubin | 0.43 | 1.45E-15 |
| 2 | 234637192 | | Direct bilirubin | 0.43 | 1.49E-15 |
| 2 | 234650604 | | Direct bilirubin | 0.43 | 1.54E-15 |
| 2 | 234548334 | | Direct bilirubin | -0.38 | 1.80E-15 |
| 2 | 234578762 | | Indirect bilirubin | -0.75 | 1.83E-15 |
| 2 | 234637912 | | Indirect bilirubin | 1.73 | 1.83E-15 |
| 2 | 234578762 | | Direct bilirubin | -0.42 | 1.90E-15 |
| 2 | 234620272 | | Total bilirubin | 1.08 | 1.93E-15 |
| 2 | 234630443 | | Indirect bilirubin | 0.98 | 2.08E-15 |
| 2 | 234534648 | | Direct bilirubin | -0.38 | 2.21E-15 |
| 2 | 234617236 | | Total bilirubin | 1.08 | 2.23E-15 |
| 2 | 234658250 | | Direct bilirubin | 0.43 | 2.31E-15 |
| 2 | 234572783 | | Direct bilirubin | -0.38 | 2.32E-15 |
| 6 | 160863702 | | Lipoprotein | 106.25 | 2.37E-15 |
| 2 | 234643749 | | Direct bilirubin | 0.42 | 2.44E-15 |
| 2 | 234637569 | | Direct bilirubin | 0.42 | 2.51E-15 |
| 2 | 234643560 | | Direct bilirubin | 0.93 | 2.56E-15 |
| 2 | 234512976 | | Direct bilirubin | -0.38 | 2.82E-15 |
| 2 | 234635241 | | Direct bilirubin | 0.42 | 3.07E-15 |
| 2 | 234635241 | | Total bilirubin | 1.46 | 3.13E-15 |
| 2 | 234578762 | | Direct bilirubin | -0.37 | 3.30E-15 |
| 2 | 234644421 | | Direct bilirubin | 0.43 | 3.33E-15 |
| 2 | 234635467 | | Total bilirubin | 1.07 | 3.40E-15 |
| 2 | 234650193 | | Total bilirubin | 1.08 | 3.51E-15 |
| 2 | 234625936 | | Indirect bilirubin | 1.54 | 3.81E-15 |
| 2 | 234622429 | | Indirect bilirubin | 0.73 | 3.89E-15 |
| 2 | 234658250 | | Indirect bilirubin | 0.98 | 4.01E-15 |
| 2 | 234617236 | | Indirect bilirubin | 0.95 | 4.12E-15 |
| 2 | 234637853 | | Total bilirubin | 1.08 | 4.37E-15 |
| 2 | 234638249 | | Direct bilirubin | 0.42 | 4.64E-15 |
| 2 | 234610912 | | Total bilirubin | 1.07 | 4.74E-15 |
| 2 | 234630443 | | Direct bilirubin | 0.42 | 4.86E-15 |
| 2 | 234647478 | | Total bilirubin | 1.07 | 5.25E-15 |
| 2 | 234673462 | | Total bilirubin | 1.08 | 5.33E-15 |
| 2 | 234650604 | | Indirect bilirubin | 0.95 | 5.38E-15 |
| 2 | 234537447 | | Direct bilirubin | -0.38 | 5.45E-15 |
| 2 | 234666461 | | Direct bilirubin | 0.42 | 5.54E-15 |
| 2 | 234625936 | | Total bilirubin | 2.32 | 6.58E-15 |
| 2 | 234635241 | | Indirect bilirubin | 0.94 | 6.82E-15 |
| 2 | 234638249 | | Direct bilirubin | 0.53 | 7.26E-15 |
| 2 | 234565438 | | Direct bilirubin | -0.37 | 7.31E-15 |
| 2 | 234634916 | | Total bilirubin | 1.07 | 7.37E-15 |
| 2 | 234637912 | | Total bilirubin | 2.58 | 7.78E-15 |
| 2 | 234550970 | | Direct bilirubin | -0.37 | 7.84E-15 |
| 2 | 234647478 | | Direct bilirubin | 0.53 | 9.20E-15 |
| 2 | 234634916 | | Indirect bilirubin | 0.96 | 9.57E-15 |
| 2 | 234637569 | | Total bilirubin | 1.07 | 1.02E-14 |
| 2 | 234628529 | | Total bilirubin | 1.06 | 1.06E-14 |
| 2 | 234513720 | | Total bilirubin | -1.10 | 1.08E-14 |
| 2 | 234512976 | | Total bilirubin | -1.09 | 1.10E-14 |
| 2 | 234666461 | | Total bilirubin | 1.06 | 1.19E-14 |
| 2 | 234655303 | | Indirect bilirubin | 0.72 | 1.19E-14 |
| 2 | 234612690 | | Total bilirubin | 1.46 | 1.22E-14 |
| 2 | 234655303 | | Total bilirubin | 1.45 | 1.26E-14 |
| 2 | 234665659 | | Direct bilirubin | 0.54 | 1.31E-14 |
| 2 | 234638690 | | Direct bilirubin | 0.54 | 1.35E-14 |
| 2 | 234583490 | | Total bilirubin | 1.00 | 1.39E-14 |
| 2 | 234695224 | | Total bilirubin | -1.63 | 1.45E-14 |
| 2 | 234583846 | | Total bilirubin | 1.00 | 1.47E-14 |
| 2 | 234630443 | | Total bilirubin | 1.06 | 1.49E-14 |
| 2 | 234628529 | | Total bilirubin | 1.43 | 1.56E-14 |
| 2 | 234634916 | | Total bilirubin | 1.45 | 1.57E-14 |
| 2 | 234637707 | | Total bilirubin | 1.07 | 1.63E-14 |
| 2 | 234653192 | | Indirect bilirubin | 0.72 | 1.69E-14 |
| 2 | 234665659 | | Indirect bilirubin | 0.72 | 1.70E-14 |
| 2 | 234643749 | | Total bilirubin | 1.05 | 1.70E-14 |
| 2 | 234653184 | | Indirect bilirubin | 0.72 | 1.78E-14 |
| 2 | 234650604 | | Total bilirubin | 1.05 | 1.90E-14 |
| 2 | 234641908 | | Direct bilirubin | 0.53 | 1.92E-14 |
| 2 | 234637192 | | Total bilirubin | 1.05 | 1.92E-14 |
| 2 | 234610539 | | Total bilirubin | 1.05 | 2.01E-14 |
| 2 | 234692539 | | Total bilirubin | -1.62 | 2.07E-14 |
| 2 | 234644421 | | Total bilirubin | 1.06 | 2.12E-14 |
| 1 | 11942856 | | Phosphorus | 2.57 | 2.16E-14 |
| 2 | 234648860 | | Direct bilirubin | 0.88 | 2.28E-14 |
| 2 | 234581920 | | Total bilirubin | 0.98 | 2.40E-14 |
| 2 | 234635241 | | Total bilirubin | 1.05 | 2.55E-14 |
| 2 | 234695224 | | Indirect bilirubin | -1.07 | 2.71E-14 |
| 2 | 234638249 | | Total bilirubin | 1.06 | 2.80E-14 |
| 2 | 234620272 | | Indirect bilirubin | 0.70 | 2.83E-14 |
| 2 | 234513720 | | Indirect bilirubin | -0.71 | 2.87E-14 |
| 2 | 234658250 | | Total bilirubin | 1.05 | 2.95E-14 |
| 2 | 234617236 | | Indirect bilirubin | 0.70 | 3.30E-14 |
| 2 | 234612690 | | Indirect bilirubin | 0.94 | 3.46E-14 |
| 2 | 234624286 | | Total bilirubin | 2.33 | 3.63E-14 |
| 2 | 234529391 | | Direct bilirubin | -0.37 | 3.98E-14 |
| 2 | 234666461 | | Indirect bilirubin | 0.70 | 4.44E-14 |
| 2 | 234694177 | | Total bilirubin | -1.60 | 4.51E-14 |
| 2 | 234683038 | | Total bilirubin | -1.61 | 4.58E-14 |
| 2 | 234512976 | | Indirect bilirubin | -0.70 | 4.98E-14 |
| 2 | 234624286 | | Indirect bilirubin | 1.52 | 5.12E-14 |
| 2 | 234689751 | | Total bilirubin | -1.59 | 5.16E-14 |
| 2 | 234635467 | | Indirect bilirubin | 0.70 | 5.22E-14 |
| 2 | 234637192 | | Direct bilirubin | 0.52 | 5.62E-14 |
| 2 | 234635467 | | Direct bilirubin | 0.51 | 5.67E-14 |
| 2 | 234637803 | | Direct bilirubin | 0.52 | 6.09E-14 |
| 2 | 234650193 | | Direct bilirubin | 0.51 | 6.14E-14 |
| 2 | 234647478 | | Indirect bilirubin | 0.70 | 6.32E-14 |
| 2 | 234512005 | | Direct bilirubin | -0.36 | 6.40E-14 |
| 2 | 234653184 | | Direct bilirubin | 0.52 | 6.69E-14 |
| 2 | 234653192 | | Direct bilirubin | 0.52 | 6.69E-14 |
| 2 | 234691428 | | Total bilirubin | -1.59 | 6.81E-14 |
| 2 | 234610912 | | Indirect bilirubin | 0.70 | 6.90E-14 |
| 2 | 234637853 | | Total bilirubin | 1.40 | 7.05E-14 |
| 2 | 234572783 | | Total bilirubin | -1.08 | 8.63E-14 |
| 2 | 234637569 | | Indirect bilirubin | 0.70 | 8.93E-14 |
| 2 | 234534648 | | Total bilirubin | -1.08 | 9.15E-14 |
| 2 | 234602641 | | Total bilirubin | 0.97 | 9.23E-14 |
| 2 | 234637853 | | Indirect bilirubin | 0.70 | 9.53E-14 |
| 2 | 234634916 | | Indirect bilirubin | 0.70 | 9.58E-14 |
| 2 | 234525355 | | Total bilirubin | -1.07 | 9.64E-14 |
| 2 | 234579892 | | Total bilirubin | 0.97 | 9.82E-14 |
| 2 | 234628529 | | Indirect bilirubin | 0.91 | 1.03E-13 |
| 2 | 234692539 | | Indirect bilirubin | -1.04 | 1.04E-13 |
| 2 | 234683038 | | Indirect bilirubin | -1.04 | 1.06E-13 |
| 2 | 234693769 | | Total bilirubin | -1.63 | 1.14E-13 |
| 2 | 234673462 | | Indirect bilirubin | 0.69 | 1.20E-13 |
| 2 | 234630443 | | Indirect bilirubin | 0.69 | 1.23E-13 |
| 2 | 234694177 | | Indirect bilirubin | -1.04 | 1.24E-13 |
| 2 | 234529391 | | Total bilirubin | -1.07 | 1.24E-13 |
| 2 | 234610912 | | Total bilirubin | 1.41 | 1.30E-13 |
| 2 | 234549226 | | Total bilirubin | 0.96 | 1.33E-13 |
| 2 | 234655303 | | Indirect bilirubin | 0.91 | 1.36E-13 |
| 2 | 234637569 | | Direct bilirubin | 0.50 | 1.42E-13 |
| 2 | 234637707 | | Direct bilirubin | 0.50 | 1.47E-13 |
| 2 | 234644421 | | Direct bilirubin | 0.50 | 1.56E-13 |
| 2 | 46571017 | | Altitude | -120.41 | 1.64E-13 |
| 2 | 234602641 | | DBIL/TBIL ratio | -0.01 | 1.77E-13 |
| 2 | 234612690 | | Direct bilirubin | 0.39 | 1.78E-13 |
| 2 | 46569770 | | Altitude | -120.30 | 1.80E-13 |
| 2 | 234583846 | | DBIL/TBIL ratio | -0.01 | 1.83E-13 |
| 2 | 234650193 | | Indirect bilirubin | 0.68 | 1.84E-13 |
| 2 | 234552292 | | Total bilirubin | -1.04 | 1.87E-13 |
| 2 | 234612690 | | Total bilirubin | 1.01 | 1.91E-13 |
| 2 | 234681416 | | Total bilirubin | -1.56 | 1.94E-13 |
| 2 | 234610539 | | Indirect bilirubin | 0.68 | 1.99E-13 |
| 2 | 234628529 | | Indirect bilirubin | 0.68 | 2.00E-13 |
| 2 | 234637707 | | Indirect bilirubin | 0.69 | 2.09E-13 |
| 2 | 234643749 | | Indirect bilirubin | 0.68 | 2.16E-13 |
| 2 | 234529391 | | Indirect bilirubin | -0.69 | 2.20E-13 |
| 2 | 234691428 | | Indirect bilirubin | -1.03 | 2.21E-13 |
| 6 | 161078184 | | Lipoprotein | 96.02 | 2.33E-13 |
| 2 | 234622429 | | Direct bilirubin | 0.52 | 2.41E-13 |
| 2 | 234689751 | | Indirect bilirubin | -1.02 | 2.44E-13 |
| 2 | 234637853 | | Indirect bilirubin | 0.90 | 2.48E-13 |
| 2 | 234620272 | | Direct bilirubin | 0.50 | 2.55E-13 |
| 2 | 234572783 | | Indirect bilirubin | -0.70 | 2.57E-13 |
| 2 | 234525355 | | Indirect bilirubin | -0.69 | 2.73E-13 |
| 2 | 234650604 | | Indirect bilirubin | 0.68 | 3.00E-13 |
| 2 | 46568084 | | Altitude | -117.95 | 3.12E-13 |
| 2 | 234637192 | | Indirect bilirubin | 0.68 | 3.12E-13 |
| 2 | 234527397 | | Total bilirubin | -1.05 | 3.31E-13 |
| 2 | 234690545 | | Total bilirubin | -1.55 | 3.31E-13 |
| 2 | 234579892 | | DBIL/TBIL ratio | -0.01 | 3.37E-13 |
| 2 | 234658250 | | Direct bilirubin | 0.51 | 3.49E-13 |
| 2 | 234693769 | | Indirect bilirubin | -1.05 | 3.53E-13 |
| 6 | 160615537 | | Lipoprotein | 114.16 | 3.60E-13 |
| 2 | 234643749 | | Direct bilirubin | 0.51 | 3.90E-13 |
| 2 | 234638249 | | Indirect bilirubin | 0.69 | 4.00E-13 |
| 2 | 234635241 | | Indirect bilirubin | 0.68 | 4.10E-13 |
| 2 | 234610912 | | Indirect bilirubin | 0.90 | 4.85E-13 |
| 2 | 234644421 | | Indirect bilirubin | 0.68 | 5.08E-13 |
| 2 | 234534648 | | Indirect bilirubin | -0.69 | 5.11E-13 |
| 2 | 234534648 | | Direct bilirubin | -0.39 | 5.20E-13 |
| 2 | 234512005 | | Total bilirubin | -1.05 | 5.29E-13 |
| 2 | 46577251 | | Altitude | -120.05 | 5.31E-13 |
| 2 | 234552292 | | Indirect bilirubin | -0.67 | 5.39E-13 |
| 2 | 234512976 | | Direct bilirubin | -0.38 | 5.48E-13 |
| 2 | 234681416 | | Indirect bilirubin | -1.01 | 5.53E-13 |
| 2 | 234513720 | | Direct bilirubin | -0.38 | 5.89E-13 |
| 2 | 234658250 | | Indirect bilirubin | 0.67 | 6.27E-13 |
| 2 | 234147256 | | Direct bilirubin | 0.85 | 6.31E-13 |
| 2 | 234149237 | | Direct bilirubin | 0.85 | 6.78E-13 |
| 2 | 46598025 | | Altitude | -116.47 | 7.01E-13 |
| 2 | 46579689 | | Altitude | -119.12 | 7.18E-13 |
| 2 | 234683892 | | Total bilirubin | -1.45 | 7.61E-13 |
| 2 | 234673462 | | Direct bilirubin | 0.50 | 7.63E-13 |
| 2 | 234683892 | | Indirect bilirubin | -0.96 | 7.79E-13 |
| 2 | 234548576 | | Total bilirubin | -1.03 | 8.00E-13 |
| 2 | 234630443 | | Direct bilirubin | 0.50 | 8.46E-13 |
| 2 | 234650604 | | Direct bilirubin | 0.49 | 8.74E-13 |
| 2 | 234690545 | | Indirect bilirubin | -1.00 | 9.00E-13 |
| 6 | 161110614 | | Lipoprotein | 93.99 | 9.83E-13 |
| 6 | 160995956 | | Lipoprotein | 83.60 | 1.13E-12 |
| 2 | 234527397 | | Indirect bilirubin | -0.68 | 1.20E-12 |
| 2 | 234548334 | | Total bilirubin | -1.03 | 1.23E-12 |
| 17 | 38149350 | | White blood cell count | -0.61 | 1.23E-12 |
| 17 | 38143532 | | White blood cell count | -0.61 | 1.30E-12 |
| 2 | 234695224 | | Direct bilirubin | -0.56 | 1.32E-12 |
| 2 | 46589323 | | Altitude | -120.66 | 1.33E-12 |
| 2 | 234643560 | | Indirect bilirubin | 1.16 | 1.47E-12 |
| 2 | 234525355 | | Direct bilirubin | -0.38 | 1.49E-12 |
| 2 | 46593536 | | Altitude | -115.28 | 1.54E-12 |
| 6 | 161009278 | | Lipoprotein | 92.66 | 1.56E-12 |
| 2 | 46594122 | | Altitude | -116.18 | 1.67E-12 |
| 6 | 160988675 | | Lipoprotein | 84.33 | 1.68E-12 |
| 2 | 234583490 | | DBIL/TBIL ratio | -0.01 | 1.69E-12 |
| 2 | 234168042 | | Direct bilirubin | 0.84 | 1.73E-12 |
| 2 | 234612690 | | Indirect bilirubin | 0.66 | 1.75E-12 |
| 2 | 234624286 | | Indirect bilirubin | 1.11 | 1.75E-12 |
| 2 | 46567916 | | Altitude | -114.44 | 1.84E-12 |
| 2 | 234548576 | | Indirect bilirubin | -0.67 | 1.85E-12 |
| 2 | 234535007 | | Total bilirubin | -1.02 | 1.86E-12 |
| 2 | 234689751 | | Direct bilirubin | -0.55 | 1.97E-12 |
| 17 | 38162983 | | White blood cell count | -0.60 | 2.03E-12 |
| 17 | 38144241 | | White blood cell count | -0.60 | 2.05E-12 |
| 2 | 234692539 | | Direct bilirubin | -0.55 | 2.11E-12 |
| 2 | 234617236 | | Direct bilirubin | 0.48 | 2.16E-12 |
| 2 | 234572783 | | Direct bilirubin | -0.38 | 2.18E-12 |
| 17 | 38153554 | | White blood cell count | -0.60 | 2.20E-12 |
| 2 | 234683038 | | Direct bilirubin | -0.55 | 2.52E-12 |
| 17 | 38144116 | | White blood cell count | -0.60 | 2.56E-12 |
| 2 | 234655303 | | Direct bilirubin | 0.49 | 2.64E-12 |
| 2 | 234694177 | | Direct bilirubin | -0.55 | 2.67E-12 |
| 2 | 234565438 | | Total bilirubin | -1.01 | 2.72E-12 |
| 2 | 46568680 | | Altitude | -113.71 | 2.74E-12 |
| 6 | 160652858 | | Lipoprotein | 78.41 | 2.79E-12 |
| 2 | 46576918 | | Altitude | -116.49 | 2.85E-12 |
| 2 | 234529391 | | Direct bilirubin | -0.37 | 2.91E-12 |
| 2 | 234691428 | | Direct bilirubin | -0.55 | 2.93E-12 |
| 17 | 38151014 | | White blood cell count | -0.60 | 2.93E-12 |
| 6 | 160649726 | | Lipoprotein | 79.10 | 3.07E-12 |
| 2 | 234548334 | | Indirect bilirubin | -0.66 | 3.24E-12 |
| 2 | 46588331 | | Altitude | -117.14 | 3.24E-12 |
| 2 | 234512005 | | Indirect bilirubin | -0.66 | 3.27E-12 |
| 2 | 234527397 | | Direct bilirubin | -0.37 | 3.55E-12 |
| 2 | 234693769 | | Direct bilirubin | -0.57 | 3.70E-12 |
| 17 | 38147657 | | White blood cell count | -0.60 | 3.88E-12 |
| 2 | 46588019 | | Altitude | -117.21 | 3.95E-12 |
| 2 | 234535007 | | Indirect bilirubin | -0.66 | 3.98E-12 |
| 17 | 38171294 | | White blood cell count | -0.59 | 4.12E-12 |
| 2 | 234552292 | | Direct bilirubin | -0.36 | 4.41E-12 |
| 17 | 38159744 | | White blood cell count | -0.59 | 4.52E-12 |
| 17 | 38159745 | | White blood cell count | -0.59 | 4.52E-12 |
| 2 | 46600358 | | Altitude | -112.83 | 4.58E-12 |
| 2 | 234635241 | | Direct bilirubin | 0.47 | 4.61E-12 |
| 17 | 38147294 | | White blood cell count | -0.59 | 5.88E-12 |
| 17 | 38174167 | | White blood cell count | -0.59 | 6.37E-12 |
| 2 | 234652740 | | Total bilirubin | -1.58 | 6.48E-12 |
| 2 | 234164492 | | Direct bilirubin | 0.80 | 7.03E-12 |
| 2 | 234233593 | | Direct bilirubin | 0.73 | 7.06E-12 |
| 2 | 234550970 | | Total bilirubin | -0.99 | 7.25E-12 |
| 2 | 234208045 | | Direct bilirubin | 0.81 | 7.27E-12 |
| 17 | 38161507 | | White blood cell count | -0.59 | 7.88E-12 |
| 17 | 38149411 | | White blood cell count | -0.58 | 8.36E-12 |
| 2 | 234681416 | | Direct bilirubin | -0.54 | 8.37E-12 |
| 2 | 234695224 | | DBIL/TBIL ratio | 0.02 | 8.73E-12 |
| 2 | 234565438 | | Indirect bilirubin | -0.65 | 8.97E-12 |
| 2 | 234185759 | | Direct bilirubin | 0.80 | 9.14E-12 |
| 17 | 38149727 | | White blood cell count | -0.59 | 9.52E-12 |
| 17 | 38176256 | | White blood cell count | -0.59 | 9.68E-12 |
| 8 | 104380925 | | Thyroid stimulating hormone | 0.92 | 9.71E-12 |
| 2 | 234637912 | | Indirect bilirubin | 1.11 | 1.00E-11 |
| 2 | 234504098 | | Direct bilirubin | -0.32 | 1.08E-11 |
| 2 | 234216917 | | Direct bilirubin | 0.73 | 1.13E-11 |
| 6 | 161123413 | | Lipoprotein | 70.41 | 1.17E-11 |
| 1 | 109815368 | | Low density lipoprotein | -0.36 | 1.23E-11 |
| 2 | 234234856 | | Direct bilirubin | 0.71 | 1.24E-11 |
| 2 | 234694177 | | DBIL/TBIL ratio | 0.02 | 1.26E-11 |
| 17 | 38149350 | | Neutrophil count | -0.54 | 1.29E-11 |
| 1 | 109815074 | | Low density lipoprotein | -0.36 | 1.30E-11 |
| 2 | 234224643 | | Direct bilirubin | 0.75 | 1.35E-11 |
| 2 | 234625936 | | Direct bilirubin | 0.75 | 1.35E-11 |
| 2 | 46597756 | | Altitude | -110.14 | 1.37E-11 |
| 2 | 46592807 | | Altitude | -110.01 | 1.39E-11 |
| 17 | 38143768 | | White blood cell count | -0.58 | 1.40E-11 |
| 2 | 234634916 | | Direct bilirubin | 0.47 | 1.42E-11 |
| 2 | 234550970 | | Indirect bilirubin | -0.64 | 1.42E-11 |
| 6 | 160635304 | | Lipoprotein | 77.09 | 1.44E-11 |
| 2 | 234628529 | | Direct bilirubin | 0.46 | 1.49E-11 |
| 2 | 234648860 | | Indirect bilirubin | 1.09 | 1.51E-11 |
| 2 | 234166007 | | Direct bilirubin | 0.79 | 1.55E-11 |
| 2 | 234605835 | | Direct bilirubin | 0.38 | 1.57E-11 |
| 17 | 38162983 | | Neutrophil count | -0.54 | 1.59E-11 |
| 2 | 234652740 | | Indirect bilirubin | -1.03 | 1.62E-11 |
| 2 | 234210329 | | Direct bilirubin | 0.54 | 1.63E-11 |
| 17 | 38153554 | | Neutrophil count | -0.54 | 1.66E-11 |
| 2 | 234637912 | | Direct bilirubin | 0.83 | 1.67E-11 |
| 2 | 234643560 | | Total bilirubin | 1.62 | 1.69E-11 |
| 2 | 234690545 | | Direct bilirubin | -0.53 | 1.77E-11 |
| 2 | 234683038 | | DBIL/TBIL ratio | 0.02 | 1.82E-11 |
| 2 | 234616357 | | Total bilirubin | 1.32 | 1.94E-11 |
| 2 | 46581643 | | Altitude | -117.63 | 1.94E-11 |
| 2 | 234624286 | | Total bilirubin | 1.56 | 1.96E-11 |
| 17 | 38143532 | | Neutrophil count | -0.53 | 1.99E-11 |
| 1 | 109815133 | | Low density lipoprotein | -0.36 | 1.99E-11 |
| 2 | 234624286 | | Direct bilirubin | 0.76 | 2.00E-11 |
| 17 | 38188419 | | White blood cell count | -0.58 | 2.03E-11 |
| 1 | 109820919 | | Low density lipoprotein | -0.36 | 2.04E-11 |
| 2 | 234548334 | | Direct bilirubin | -0.36 | 2.14E-11 |
| 2 | 234548576 | | Direct bilirubin | -0.36 | 2.19E-11 |
| 17 | 38186825 | | White blood cell count | -0.58 | 2.26E-11 |
| 2 | 234512005 | | Direct bilirubin | -0.36 | 2.44E-11 |
| 6 | 161122763 | | Lipoprotein | 68.45 | 2.50E-11 |
| 17 | 38147657 | | Neutrophil count | -0.53 | 2.55E-11 |
| 17 | 38151014 | | Neutrophil count | -0.53 | 2.56E-11 |
| 17 | 38180352 | | White blood cell count | -0.57 | 2.67E-11 |
| 2 | 234171459 | | Direct bilirubin | 0.77 | 2.80E-11 |
| 2 | 234565438 | | Direct bilirubin | -0.36 | 2.81E-11 |
| 2 | 234693769 | | DBIL/TBIL ratio | 0.02 | 2.88E-11 |
| 2 | 234201700 | | Direct bilirubin | 0.84 | 2.90E-11 |
| 2 | 234691428 | | DBIL/TBIL ratio | 0.02 | 2.97E-11 |
| 17 | 38144116 | | Neutrophil count | -0.53 | 3.00E-11 |
| 2 | 234619937 | | Total bilirubin | 1.33 | 3.05E-11 |
| 2 | 234681416 | | DBIL/TBIL ratio | 0.02 | 3.11E-11 |
| 1 | 109815746 | | Low density lipoprotein | -0.36 | 3.13E-11 |
| 1 | 109815211 | | Low density lipoprotein | -0.36 | 3.19E-11 |
| 2 | 234612690 | | Direct bilirubin | 0.46 | 3.27E-11 |
| 2 | 46577797 | | Altitude | -108.73 | 3.32E-11 |
| 17 | 38144241 | | Neutrophil count | -0.53 | 3.34E-11 |
| 2 | 234395159 | | Total bilirubin | -1.16 | 3.34E-11 |
| 2 | 234429527 | | Indirect bilirubin | 1.05 | 3.71E-11 |
| 2 | 234676872 | | Direct bilirubin | 0.52 | 3.89E-11 |
| 17 | 38159744 | | Neutrophil count | -0.53 | 4.08E-11 |
| 17 | 38159745 | | Neutrophil count | -0.53 | 4.08E-11 |
| 2 | 234535007 | | Direct bilirubin | -0.35 | 4.10E-11 |
| 2 | 234687371 | | Direct bilirubin | 0.52 | 4.38E-11 |
| 2 | 234683892 | | Direct bilirubin | -0.49 | 4.45E-11 |
| 2 | 234643560 | | Direct bilirubin | 0.61 | 4.53E-11 |
| 17 | 38171294 | | Neutrophil count | -0.52 | 4.58E-11 |
| 17 | 38174167 | | Neutrophil count | -0.53 | 4.61E-11 |
| 17 | 38147294 | | Neutrophil count | -0.52 | 4.64E-11 |
| 2 | 234690545 | | DBIL/TBIL ratio | 0.02 | 4.64E-11 |
| 2 | 234207080 | | Direct bilirubin | 0.89 | 5.07E-11 |
| 17 | 38189049 | | White blood cell count | -0.56 | 5.30E-11 |
| 2 | 234234922 | | Direct bilirubin | 0.70 | 5.41E-11 |
| 2 | 46577808 | | Altitude | -107.62 | 5.58E-11 |
| 17 | 38167627 | | White blood cell count | -0.54 | 5.66E-11 |
| 17 | 38196957 | | White blood cell count | -0.56 | 5.69E-11 |
| 17 | 38149727 | | Neutrophil count | -0.52 | 5.75E-11 |
| 2 | 234624286 | | Direct bilirubin | 0.59 | 6.62E-11 |
| 2 | 234610477 | | Direct bilirubin | 0.54 | 6.89E-11 |
| 2 | 234210329 | | Total bilirubin | 1.40 | 7.10E-11 |
| 2 | 234149237 | | Total bilirubin | 2.09 | 7.17E-11 |
| 17 | 38176256 | | Neutrophil count | -0.52 | 7.22E-11 |
| 2 | 234616357 | | Direct bilirubin | 0.37 | 7.47E-11 |
| 2 | 46567227 | | Altitude | -108.34 | 7.53E-11 |
| 17 | 38149411 | | Neutrophil count | -0.52 | 7.58E-11 |
| 17 | 38143768 | | Neutrophil count | -0.52 | 7.68E-11 |
| 2 | 233243823 | | Alkaline phosphatase | -24.13 | 7.77E-11 |
| 2 | 234233593 | | Total bilirubin | 1.86 | 8.03E-11 |
| 2 | 233244223 | | Alkaline phosphatase | -24.14 | 8.11E-11 |
| 2 | 233243334 | | Alkaline phosphatase | -23.93 | 8.20E-11 |
| 2 | 46601112 | | Altitude | -113.04 | 8.80E-11 |
| 6 | 160674557 | | Lipoprotein | 70.28 | 9.14E-11 |
| 2 | 233242774 | | Alkaline phosphatase | -22.40 | 9.92E-11 |
| 2 | 234193663 | | Direct bilirubin | 0.75 | 1.01E-10 |
| 2 | 234429527 | | Total bilirubin | 1.57 | 1.01E-10 |
| 6 | 161121210 | | Lipoprotein | 66.36 | 1.03E-10 |
| 17 | 38161507 | | Neutrophil count | -0.52 | 1.03E-10 |
| 17 | 38157021 | | White blood cell count | -0.54 | 1.05E-10 |
| 2 | 234234856 | | Total bilirubin | 1.83 | 1.06E-10 |
| 2 | 234395159 | | Indirect bilirubin | -0.75 | 1.12E-10 |
| 2 | 234201658 | | Direct bilirubin | 0.80 | 1.18E-10 |
| 2 | 234637912 | | Total bilirubin | 1.54 | 1.22E-10 |
| 6 | 160850273 | | Lipoprotein | 47.55 | 1.24E-10 |
| 11 | 116606766 | | Triglyceride | 0.11 | 1.26E-10 |
| 17 | 38164851 | | White blood cell count | -0.53 | 1.28E-10 |
| 2 | 234147256 | | Total bilirubin | 2.07 | 1.33E-10 |
| 2 | 234227118 | | Direct bilirubin | 0.69 | 1.34E-10 |
| 2 | 234357513 | | Total bilirubin | -1.12 | 1.34E-10 |
| 1 | 109817479 | | Low density lipoprotein | -0.34 | 1.42E-10 |
| 2 | 234619937 | | Direct bilirubin | 0.36 | 1.50E-10 |
| 6 | 161123771 | | Lipoprotein | 64.29 | 1.59E-10 |
| 2 | 234168042 | | Total bilirubin | 2.05 | 1.61E-10 |
| 17 | 38205396 | | White blood cell count | -0.55 | 1.68E-10 |
| 17 | 38188419 | | Neutrophil count | -0.51 | 1.69E-10 |
| 2 | 234216917 | | Total bilirubin | 1.85 | 1.69E-10 |
| 2 | 234210329 | | Direct bilirubin | 0.42 | 1.69E-10 |
| 2 | 234550970 | | Direct bilirubin | -0.34 | 1.71E-10 |
| 2 | 234637853 | | Direct bilirubin | 0.44 | 1.71E-10 |
| 6 | 161121303 | | Lipoprotein | 63.93 | 1.72E-10 |
| 2 | 234208045 | | Total bilirubin | 2.05 | 1.75E-10 |
| 1 | 109820110 | | Low density lipoprotein | -0.36 | 1.76E-10 |
| 2 | 234648860 | | Total bilirubin | 1.51 | 1.78E-10 |
| 2 | 234692539 | | DBIL/TBIL ratio | 0.02 | 1.82E-10 |
| 2 | 234233066 | | Direct bilirubin | 0.71 | 1.83E-10 |
| 2 | 234154614 | | Direct bilirubin | 0.74 | 1.86E-10 |
| 17 | 38180352 | | Neutrophil count | -0.51 | 1.92E-10 |
| 6 | 161119901 | | Lipoprotein | 64.54 | 1.96E-10 |
| 2 | 46581732 | | Altitude | -109.95 | 1.98E-10 |
| 2 | 234224643 | | Total bilirubin | 1.90 | 1.99E-10 |
| 2 | 234491647 | | Indirect bilirubin | 1.01 | 2.00E-10 |
| 2 | 234395159 | | Direct bilirubin | -0.41 | 2.06E-10 |
| 2 | 233246489 | | Alkaline phosphatase | -23.53 | 2.12E-10 |
| 2 | 234616357 | | Indirect bilirubin | 0.82 | 2.12E-10 |
| 2 | 234652740 | | Direct bilirubin | -0.54 | 2.12E-10 |
| 2 | 234357513 | | Direct bilirubin | -0.41 | 2.15E-10 |
| 2 | 234637912 | | Direct bilirubin | 0.58 | 2.17E-10 |
| 2 | 233252671 | | Alkaline phosphatase | -23.32 | 2.24E-10 |
| 2 | 234551777 | | Direct bilirubin | 0.51 | 2.29E-10 |
| 2 | 234610912 | | Direct bilirubin | 0.45 | 2.38E-10 |
| 2 | 234619937 | | Indirect bilirubin | 0.83 | 2.44E-10 |
| 2 | 234234538 | | Direct bilirubin | 0.67 | 2.49E-10 |
| 6 | 160695773 | | Lipoprotein | -44.22 | 2.51E-10 |
| 17 | 38186825 | | Neutrophil count | -0.51 | 2.52E-10 |
| 2 | 234625936 | | Indirect bilirubin | 1.02 | 2.64E-10 |
| 2 | 234578762 | | DBIL/TBIL ratio | 0.01 | 2.68E-10 |
| 2 | 234617365 | | Direct bilirubin | 0.36 | 2.74E-10 |
| 2 | 234185759 | | Total bilirubin | 1.99 | 2.75E-10 |
| 2 | 46630568 | | Altitude | -107.17 | 2.81E-10 |
| 2 | 234617365 | | Total bilirubin | 1.26 | 2.85E-10 |
| 2 | 234689751 | | DBIL/TBIL ratio | 0.02 | 3.06E-10 |
| 2 | 234605835 | | Total bilirubin | 1.28 | 3.08E-10 |
| 2 | 234357513 | | Indirect bilirubin | -0.73 | 3.19E-10 |
| 17 | 38168227 | | White blood cell count | -0.52 | 3.23E-10 |
| 2 | 234683892 | | DBIL/TBIL ratio | 0.02 | 3.25E-10 |
| 2 | 46601414 | | Altitude | -107.94 | 3.31E-10 |
| 2 | 234648860 | | Direct bilirubin | 0.58 | 3.37E-10 |
| 2 | 234148244 | | Direct bilirubin | 0.79 | 3.39E-10 |
| 2 | 234464134 | | Indirect bilirubin | 1.00 | 3.40E-10 |
| 2 | 46610479 | | Altitude | -105.16 | 3.45E-10 |
| 2 | 234154936 | | Direct bilirubin | 0.73 | 3.58E-10 |
| 2 | 234512976 | | DBIL/TBIL ratio | 0.01 | 3.64E-10 |
| 2 | 234668870 | | Direct bilirubin | 0.49 | 3.77E-10 |
| 2 | 234513720 | | DBIL/TBIL ratio | 0.01 | 3.78E-10 |
| 6 | 161122931 | | Lipoprotein | 62.82 | 3.82E-10 |
| 2 | 234539111 | | Direct bilirubin | 0.38 | 3.84E-10 |
| 17 | 38196957 | | Neutrophil count | -0.50 | 3.97E-10 |
| 1 | 12009223 | | Thyroid stimulating hormone | 0.44 | 4.01E-10 |
| 2 | 234584324 | | Total bilirubin | 1.24 | 4.20E-10 |
| 2 | 234227118 | | Total bilirubin | 1.80 | 4.26E-10 |
| 2 | 234549226 | | Direct bilirubin | 0.31 | 4.30E-10 |
| 2 | 46577265 | | Altitude | -108.82 | 4.37E-10 |
| 1 | 11968356 | | Phosphorus | 1.58 | 4.80E-10 |
| 2 | 234494513 | | Indirect bilirubin | 0.96 | 4.89E-10 |
| 2 | 234381817 | | Total bilirubin | -1.09 | 4.96E-10 |
| 1 | 11968356 | | Thyroid stimulating hormone | -0.47 | 4.96E-10 |
| 9 | 107556416 | | High density lipoprotein | -0.05 | 4.99E-10 |
| 9 | 107556417 | | High density lipoprotein | -0.05 | 4.99E-10 |
| 17 | 38189049 | | Neutrophil count | -0.50 | 5.03E-10 |
| 2 | 46630374 | | Altitude | -108.32 | 5.03E-10 |
| 2 | 234548576 | | DBIL/TBIL ratio | 0.01 | 5.06E-10 |
| 2 | 234497823 | | Indirect bilirubin | 0.96 | 5.07E-10 |
| 6 | 160839350 | | Lipoprotein | 46.56 | 5.14E-10 |
| 2 | 234416178 | | Indirect bilirubin | 0.99 | 5.16E-10 |
| 17 | 38205396 | | Neutrophil count | -0.50 | 5.24E-10 |
| 17 | 38169714 | | White blood cell count | -0.51 | 5.36E-10 |
| 1 | 11942856 | | Thyroid stimulating hormone | -0.62 | 5.48E-10 |
| 2 | 46600894 | | Altitude | -105.38 | 5.68E-10 |
| 2 | 46602722 | | Altitude | -106.23 | 5.73E-10 |
| 2 | 234234922 | | Total bilirubin | 1.78 | 5.80E-10 |
| 1 | 11976219 | | Thyroid stimulating hormone | -0.47 | 5.84E-10 |
| 2 | 234450058 | | Indirect bilirubin | 0.97 | 5.95E-10 |
| 1 | 11967905 | | Thyroid stimulating hormone | -0.47 | 5.98E-10 |
| 1 | 11973842 | | Thyroid stimulating hormone | -0.47 | 6.05E-10 |
| 2 | 234702459 | | Indirect bilirubin | -0.62 | 6.21E-10 |
| 2 | 234529391 | | DBIL/TBIL ratio | 0.01 | 6.23E-10 |
| 2 | 46577299 | | Altitude | -108.59 | 6.39E-10 |
| 1 | 11986621 | | Thyroid stimulating hormone | -0.47 | 6.43E-10 |
| 1 | 11967905 | | Phosphorus | 1.57 | 6.51E-10 |
| 6 | 161123158 | | Lipoprotein | 62.38 | 6.55E-10 |
| 2 | 234584324 | | Direct bilirubin | 0.34 | 6.55E-10 |
| 2 | 234171459 | | Total bilirubin | 1.93 | 6.67E-10 |
| 6 | 160637485 | | Lipoprotein | 68.78 | 6.79E-10 |
| 2 | 46603260 | | Altitude | -107.47 | 6.82E-10 |
| 2 | 234381817 | | Indirect bilirubin | -0.71 | 6.91E-10 |
| 6 | 161117913 | | Lipoprotein | 61.39 | 7.10E-10 |
| 17 | 38161823 | | White blood cell count | -0.51 | 7.16E-10 |
| 2 | 234501740 | | Indirect bilirubin | 0.97 | 7.23E-10 |
| 2 | 46577212 | | Altitude | -108.03 | 7.36E-10 |
| 2 | 46579409 | | Altitude | -106.19 | 7.43E-10 |
| 2 | 46603165 | | Altitude | -106.91 | 7.48E-10 |
| 6 | 161120058 | | Lipoprotein | 61.46 | 7.55E-10 |
| 1 | 12009956 | | Thyroid stimulating hormone | 0.43 | 7.56E-10 |
| 6 | 160995964 | | Lipoprotein | -57.04 | 7.77E-10 |
| 2 | 234164492 | | Total bilirubin | 1.93 | 8.02E-10 |
| 2 | 234616357 | | Total bilirubin | 0.89 | 8.03E-10 |
| 1 | 11968406 | | Thyroid stimulating hormone | -0.47 | 8.16E-10 |
| 17 | 38157920 | | White blood cell count | -0.50 | 8.19E-10 |
| 1 | 12013192 | | Phosphorus | 1.99 | 8.26E-10 |
| 1 | 12013196 | | Phosphorus | 1.99 | 8.26E-10 |
| 1 | 12013197 | | Phosphorus | 1.99 | 8.26E-10 |
| 1 | 12013198 | | Phosphorus | 1.99 | 8.26E-10 |
| 1 | 12013208 | | Phosphorus | 1.99 | 8.26E-10 |
| 17 | 38169273 | | White blood cell count | -0.50 | 8.59E-10 |
| 6 | 160695270 | | Lipoprotein | -42.94 | 8.64E-10 |
| 2 | 169859806 | | Gamma-glutamyl transpeptidase | 2.40 | 8.76E-10 |
| 6 | 161123185 | | Lipoprotein | 62.98 | 8.76E-10 |
| 2 | 234551777 | | Total bilirubin | 1.80 | 8.83E-10 |
| 2 | 234687371 | | Total bilirubin | 1.23 | 9.21E-10 |
| 2 | 234552292 | | DBIL/TBIL ratio | 0.01 | 9.28E-10 |
| 2 | 234676872 | | Total bilirubin | 1.24 | 9.31E-10 |
| 2 | 46580474 | | Altitude | -105.64 | 9.47E-10 |
| 9 | 107569337 | | High density lipoprotein | -0.05 | 9.56E-10 |
| 7 | 140515399 | | Gestation | -2.00 | 9.77E-10 |
| 2 | 234525355 | | DBIL/TBIL ratio | 0.01 | 9.94E-10 |
| 8 | 104299739 | | Thyroid stimulating hormone | 0.86 | 9.99E-10 |
| 1 | 11976231 | | Thyroid stimulating hormone | -0.46 | 1.02E-09 |
| 2 | 234422821 | | Indirect bilirubin | 0.92 | 1.03E-09 |
| 2 | 46597870 | | Altitude | -105.77 | 1.03E-09 |
| 2 | 234233019 | | Direct bilirubin | 0.61 | 1.06E-09 |
| 6 | 160694081 | | Lipoprotein | -42.85 | 1.07E-09 |
| 2 | 234535007 | | DBIL/TBIL ratio | 0.01 | 1.08E-09 |
| 6 | 160696318 | | Lipoprotein | -42.82 | 1.09E-09 |
| 2 | 234625936 | | Direct bilirubin | 0.56 | 1.09E-09 |
| 2 | 234416178 | | Total bilirubin | 1.47 | 1.14E-09 |
| 2 | 234607379 | | Total bilirubin | -1.31 | 1.16E-09 |
| 2 | 234489656 | | Indirect bilirubin | 0.95 | 1.18E-09 |
| 2 | 46628545 | | Altitude | -104.10 | 1.22E-09 |
| 2 | 46616065 | | Altitude | -109.15 | 1.22E-09 |
| 2 | 234625936 | | Total bilirubin | 1.44 | 1.24E-09 |
| 2 | 234493048 | | Indirect bilirubin | 0.92 | 1.29E-09 |
| 20 | 57559630 | | Platelet distribution width | 0.09 | 1.31E-09 |
| 2 | 234493876 | | Indirect bilirubin | 0.92 | 1.32E-09 |
| 2 | 46575388 | | Altitude | -104.51 | 1.33E-09 |
| 6 | 160693055 | | Lipoprotein | -42.43 | 1.35E-09 |
| 2 | 46629712 | | Altitude | -104.45 | 1.48E-09 |
| 17 | 38213443 | | White blood cell count | -0.50 | 1.50E-09 |
| 2 | 234255145 | | Total bilirubin | -1.02 | 1.52E-09 |
| 2 | 234500532 | | Indirect bilirubin | 0.94 | 1.53E-09 |
| 2 | 234298826 | | Total bilirubin | -1.05 | 1.56E-09 |
| 2 | 234166007 | | Total bilirubin | 1.91 | 1.57E-09 |
| 17 | 38169095 | | White blood cell count | -0.50 | 1.61E-09 |
| 2 | 46601496 | | Altitude | -102.70 | 1.62E-09 |
| 2 | 234617365 | | Total bilirubin | 0.87 | 1.63E-09 |
| 7 | 140618778 | | Gestation | -2.01 | 1.63E-09 |
| 2 | 234551777 | | Indirect bilirubin | 1.15 | 1.64E-09 |
| 2 | 234157772 | | Direct bilirubin | 0.79 | 1.64E-09 |
| 2 | 234451882 | | Indirect bilirubin | 0.91 | 1.65E-09 |
| 2 | 234455344 | | Indirect bilirubin | 0.92 | 1.66E-09 |
| 2 | 234471832 | | Indirect bilirubin | 0.93 | 1.68E-09 |
| 11 | 125806207 | | Hemoglobin | -4.53 | 1.69E-09 |
| 2 | 234255145 | | Indirect bilirubin | -0.67 | 1.69E-09 |
| 2 | 234508768 | | Total bilirubin | 1.50 | 1.70E-09 |
| 2 | 234616357 | | Direct bilirubin | 0.44 | 1.70E-09 |
| 2 | 234201700 | | Total bilirubin | 2.06 | 1.71E-09 |
| 2 | 234418427 | | Indirect bilirubin | 0.91 | 1.73E-09 |
| 2 | 46603438 | | Altitude | -105.38 | 1.75E-09 |
| 2 | 234572783 | | DBIL/TBIL ratio | 0.01 | 1.77E-09 |
| 2 | 234513372 | | Total bilirubin | 1.52 | 1.77E-09 |
| 1 | 11968273 | | Phosphorus | 1.54 | 1.77E-09 |
| 2 | 234278549 | | Indirect bilirubin | -0.67 | 1.78E-09 |
| 17 | 38153875 | | White blood cell count | -0.50 | 1.79E-09 |
| 2 | 46624723 | | Altitude | -103.86 | 1.80E-09 |
| 2 | 233269060 | | Alkaline phosphatase | -23.77 | 1.81E-09 |
| 9 | 107554069 | | High density lipoprotein | -0.04 | 1.85E-09 |
| 2 | 234204672 | | Total bilirubin | -0.86 | 1.92E-09 |
| 2 | 234201658 | | Total bilirubin | 2.03 | 1.93E-09 |
| 2 | 234415925 | | Indirect bilirubin | 0.91 | 1.93E-09 |
| 1 | 12008863 | | Thyroid stimulating hormone | 0.41 | 1.93E-09 |
| 2 | 234418873 | | Indirect bilirubin | 0.94 | 1.94E-09 |
| 2 | 234605835 | | Total bilirubin | 0.86 | 1.96E-09 |
| 2 | 234539111 | | Total bilirubin | 0.93 | 1.98E-09 |
| 2 | 234402614 | | Indirect bilirubin | 0.90 | 1.99E-09 |
| 2 | 234653184 | | DBIL/TBIL ratio | -0.01 | 1.99E-09 |
| 2 | 234653192 | | DBIL/TBIL ratio | -0.01 | 1.99E-09 |
| 17 | 38215695 | | White blood cell count | -0.49 | 2.02E-09 |
| 2 | 234464134 | | Total bilirubin | 1.46 | 2.03E-09 |
| 6 | 160696828 | | Lipoprotein | -41.96 | 2.05E-09 |
| 2 | 234491647 | | Total bilirubin | 1.44 | 2.05E-09 |
| 2 | 234427312 | | Indirect bilirubin | 0.90 | 2.12E-09 |
| 6 | 161121390 | | Lipoprotein | 60.32 | 2.12E-09 |
| 2 | 234491725 | | Indirect bilirubin | 0.91 | 2.14E-09 |
| 2 | 234234538 | | Total bilirubin | 1.71 | 2.16E-09 |
| 2 | 46626828 | | Altitude | -103.41 | 2.16E-09 |
| 11 | 125814198 | | Hemoglobin | -4.36 | 2.16E-09 |
| 17 | 38167627 | | Neutrophil count | -0.46 | 2.18E-09 |
| 11 | 125799404 | | Hemoglobin | -4.47 | 2.22E-09 |
| 2 | 234142691 | | Direct bilirubin | 0.68 | 2.22E-09 |
| 2 | 234497823 | | Total bilirubin | 1.40 | 2.25E-09 |
| 2 | 234278549 | | Total bilirubin | -1.01 | 2.30E-09 |
| 2 | 234425840 | | Indirect bilirubin | 0.93 | 2.31E-09 |
| 2 | 234610477 | | Total bilirubin | 1.88 | 2.34E-09 |
| 2 | 234332439 | | Total bilirubin | 1.35 | 2.38E-09 |
| 2 | 169860564 | | Gamma-glutamyl transpeptidase | 2.26 | 2.39E-09 |
| 6 | 160695677 | | Lipoprotein | -41.56 | 2.41E-09 |
| 2 | 234512005 | | DBIL/TBIL ratio | 0.01 | 2.45E-09 |
| 2 | 234332439 | | Indirect bilirubin | 0.88 | 2.48E-09 |
| 2 | 234298826 | | Indirect bilirubin | -0.68 | 2.52E-09 |
| 2 | 234417312 | | Indirect bilirubin | 0.90 | 2.53E-09 |
| 2 | 234565438 | | DBIL/TBIL ratio | 0.01 | 2.54E-09 |
| 2 | 234420787 | | Indirect bilirubin | 0.95 | 2.54E-09 |
| 2 | 234417428 | | Indirect bilirubin | 0.91 | 2.56E-09 |
| 2 | 234166007 | | Direct bilirubin | 0.71 | 2.63E-09 |
| 2 | 46632083 | | Altitude | -102.92 | 2.63E-09 |
| 1 | 11976219 | | Phosphorus | 1.51 | 2.66E-09 |
| 20 | 57554367 | | Platelet distribution width | 0.09 | 2.67E-09 |
| 2 | 234502121 | | Indirect bilirubin | 0.88 | 2.72E-09 |
| 1 | 12008515 | | Thyroid stimulating hormone | 0.42 | 2.74E-09 |
| 2 | 234402375 | | Indirect bilirubin | 0.90 | 2.76E-09 |
| 2 | 234429527 | | Direct bilirubin | 0.54 | 2.77E-09 |
| 17 | 38188844 | | White blood cell count | -0.49 | 2.81E-09 |
| 2 | 234676872 | | Total bilirubin | 1.72 | 2.81E-09 |
| 2 | 234607379 | | Indirect bilirubin | -0.85 | 2.83E-09 |
| 6 | 161110273 | | Lipoprotein | 59.96 | 2.85E-09 |
| 2 | 234483489 | | Indirect bilirubin | 0.90 | 2.86E-09 |
| 2 | 234494513 | | Total bilirubin | 1.39 | 2.87E-09 |
| 2 | 46630434 | | Altitude | -102.51 | 2.89E-09 |
| 2 | 234193663 | | Total bilirubin | 1.86 | 2.89E-09 |
| 2 | 234551777 | | Direct bilirubin | 0.64 | 2.90E-09 |
| 2 | 234411029 | | Indirect bilirubin | 0.89 | 2.92E-09 |
| 17 | 38179290 | | White blood cell count | -0.49 | 2.92E-09 |
| 2 | 46625781 | | Altitude | -103.34 | 2.94E-09 |
| 2 | 234207080 | | Total bilirubin | 2.18 | 2.96E-09 |
| 6 | 160693906 | | Lipoprotein | -41.70 | 2.96E-09 |
| 2 | 234406021 | | Indirect bilirubin | 0.92 | 2.97E-09 |
| 2 | 234381780 | | Indirect bilirubin | 0.90 | 2.98E-09 |
| 1 | 11968406 | | Phosphorus | 1.50 | 2.98E-09 |
| 2 | 234445799 | | Indirect bilirubin | 0.88 | 3.00E-09 |
| 2 | 234233066 | | Total bilirubin | 1.78 | 3.03E-09 |
| 2 | 234584324 | | Indirect bilirubin | 0.77 | 3.03E-09 |
| 2 | 234643749 | | DBIL/TBIL ratio | -0.01 | 3.05E-09 |
| 2 | 169863060 | | Gamma-glutamyl transpeptidase | 2.27 | 3.13E-09 |
| 20 | 57556488 | | Platelet distribution width | 0.09 | 3.17E-09 |
| 2 | 234457312 | | Indirect bilirubin | 0.89 | 3.19E-09 |
| 2 | 46602678 | | Altitude | -101.57 | 3.28E-09 |
| 2 | 234460385 | | Indirect bilirubin | 0.92 | 3.28E-09 |
| 2 | 234551777 | | Total bilirubin | 1.21 | 3.30E-09 |
| 2 | 234605835 | | Indirect bilirubin | 0.79 | 3.31E-09 |
| 2 | 234617365 | | Indirect bilirubin | 0.77 | 3.33E-09 |
| 2 | 234619937 | | Total bilirubin | 0.85 | 3.33E-09 |
| 11 | 125812350 | | Hemoglobin | -4.39 | 3.38E-09 |
| 17 | 38130139 | | White blood cell count | -0.49 | 3.41E-09 |
| 2 | 234550970 | | DBIL/TBIL ratio | 0.01 | 3.41E-09 |
| 2 | 234224643 | | Direct bilirubin | 0.64 | 3.42E-09 |
| 17 | 38164851 | | Neutrophil count | -0.46 | 3.43E-09 |
| 2 | 234412079 | | Indirect bilirubin | 0.91 | 3.74E-09 |
| 1 | 11976745 | | Thyroid stimulating hormone | -0.47 | 3.78E-09 |
| 2 | 234430931 | | Indirect bilirubin | 0.90 | 3.78E-09 |
| 2 | 234246137 | | Total bilirubin | -0.84 | 3.78E-09 |
| 2 | 46599030 | | Altitude | -100.50 | 3.80E-09 |
| 1 | 11949970 | | Phosphorus | 1.65 | 3.80E-09 |
| 2 | 234483673 | | Indirect bilirubin | 0.89 | 3.81E-09 |
| 2 | 46589295 | | Altitude | -110.59 | 3.87E-09 |
| 2 | 234422821 | | Total bilirubin | 1.36 | 3.89E-09 |
| 1 | 12000632 | | Thyroid stimulating hormone | 0.42 | 3.89E-09 |
| 2 | 234224127 | | Direct bilirubin | 0.65 | 3.90E-09 |
| 20 | 57559677 | | Platelet distribution width | 0.08 | 3.91E-09 |
| 2 | 234471832 | | Total bilirubin | 1.38 | 3.92E-09 |
| 2 | 234501740 | | Total bilirubin | 1.41 | 3.95E-09 |
| 6 | 161122707 | | Lipoprotein | 59.45 | 3.96E-09 |
| 2 | 169872462 | | Gamma-glutamyl transpeptidase | 2.07 | 3.96E-09 |
| 2 | 46596433 | | Altitude | -102.72 | 4.00E-09 |
| 11 | 116580815 | | Triglyceride | 0.09 | 4.00E-09 |
| 6 | 161120648 | | Lipoprotein | 59.98 | 4.03E-09 |
| 2 | 234425840 | | Total bilirubin | 1.39 | 4.04E-09 |
| 2 | 234583846 | | Direct bilirubin | 0.30 | 4.07E-09 |
| 1 | 11976231 | | Phosphorus | 1.49 | 4.19E-09 |
| 2 | 234402411 | | Indirect bilirubin | 0.88 | 4.25E-09 |
| 17 | 38189793 | | White blood cell count | -0.48 | 4.35E-09 |
| 2 | 234475235 | | Indirect bilirubin | 0.90 | 4.36E-09 |
| 2 | 234499326 | | Indirect bilirubin | 0.89 | 4.44E-09 |
| 2 | 234241662 | | Direct bilirubin | 0.64 | 4.45E-09 |
| 2 | 234469013 | | Indirect bilirubin | 0.90 | 4.47E-09 |
| 2 | 234460385 | | Total bilirubin | 1.39 | 4.53E-09 |
| 2 | 234452015 | | Indirect bilirubin | 0.88 | 4.54E-09 |
| 2 | 234447999 | | Indirect bilirubin | 0.92 | 4.57E-09 |
| 2 | 169862936 | | Gamma-glutamyl transpeptidase | 2.24 | 4.60E-09 |
| 2 | 234400404 | | Indirect bilirubin | 0.89 | 4.61E-09 |
| 2 | 234491077 | | Indirect bilirubin | 0.88 | 4.62E-09 |
| 2 | 234668870 | | Total bilirubin | 1.18 | 4.64E-09 |
| 17 | 38203652 | | White blood cell count | -0.48 | 4.67E-09 |
| 2 | 234381817 | | Direct bilirubin | -0.38 | 4.70E-09 |
| 2 | 233260747 | | Alkaline phosphatase | -23.44 | 4.81E-09 |
| 2 | 234418427 | | Total bilirubin | 1.35 | 4.84E-09 |
| 2 | 234248110 | | Total bilirubin | -0.83 | 4.84E-09 |
| 2 | 234455213 | | Indirect bilirubin | 0.87 | 4.86E-09 |
| 2 | 234418405 | | Indirect bilirubin | 0.89 | 4.92E-09 |
| 2 | 46579273 | | Altitude | -103.71 | 4.92E-09 |
| 2 | 234637569 | | DBIL/TBIL ratio | -0.01 | 4.97E-09 |
| 2 | 234157772 | | Direct bilirubin | 0.72 | 4.98E-09 |
| 6 | 160693266 | | Lipoprotein | -40.88 | 5.01E-09 |
| 2 | 234607379 | | Direct bilirubin | -0.47 | 5.01E-09 |
| 2 | 234255145 | | Direct bilirubin | -0.37 | 5.06E-09 |
| 2 | 234412530 | | Indirect bilirubin | 0.88 | 5.10E-09 |
| 6 | 160694952 | | Lipoprotein | -40.84 | 5.14E-09 |
| 17 | 38215117 | | White blood cell count | -0.48 | 5.14E-09 |
| 2 | 234619937 | | Direct bilirubin | 0.43 | 5.15E-09 |
| 2 | 234489656 | | Total bilirubin | 1.38 | 5.20E-09 |
| 2 | 234401581 | | Indirect bilirubin | 0.87 | 5.24E-09 |
| 2 | 234421754 | | Indirect bilirubin | 0.88 | 5.32E-09 |
| 9 | 107635869 | | High density lipoprotein | -0.04 | 5.50E-09 |
| 2 | 234638249 | | DBIL/TBIL ratio | -0.01 | 5.56E-09 |
| 2 | 234450058 | | Total bilirubin | 1.39 | 5.62E-09 |
| 2 | 234420754 | | Indirect bilirubin | 0.93 | 5.63E-09 |
| 6 | 160693213 | | Lipoprotein | -40.77 | 5.63E-09 |
| 2 | 234246458 | | Direct bilirubin | -0.32 | 5.63E-09 |
| 2 | 234508692 | | Total bilirubin | 1.44 | 5.66E-09 |
| 2 | 234472396 | | Indirect bilirubin | 0.87 | 5.67E-09 |
| 2 | 234224127 | | Direct bilirubin | 0.71 | 5.74E-09 |
| 2 | 234233883 | | Direct bilirubin | 0.67 | 5.79E-09 |
| 2 | 234225681 | | Direct bilirubin | 0.59 | 5.87E-09 |
| 2 | 234468042 | | Indirect bilirubin | 0.88 | 5.89E-09 |
| 2 | 234402614 | | Total bilirubin | 1.34 | 5.92E-09 |
| 17 | 38194799 | | White blood cell count | -0.48 | 5.96E-09 |
| 2 | 234408547 | | Indirect bilirubin | 0.86 | 6.00E-09 |
| 1 | 156867984 | | Platelets | -12.01 | 6.01E-09 |
| 20 | 57557544 | | Platelet distribution width | 0.08 | 6.13E-09 |
| 2 | 234246137 | | Indirect bilirubin | -0.54 | 6.23E-09 |
| 2 | 234534648 | | DBIL/TBIL ratio | 0.01 | 6.25E-09 |
| 2 | 234418209 | | Indirect bilirubin | 0.86 | 6.32E-09 |
| 2 | 233272848 | | Alkaline phosphatase | -22.78 | 6.36E-09 |
| 1 | 11955587 | | Thyroid stimulating hormone | -0.59 | 6.44E-09 |
| 2 | 234548334 | | DBIL/TBIL ratio | 0.01 | 6.48E-09 |
| 2 | 234584324 | | Total bilirubin | 0.83 | 6.49E-09 |
| 2 | 234384293 | | Indirect bilirubin | 0.86 | 6.51E-09 |
| 2 | 234493048 | | Total bilirubin | 1.33 | 6.52E-09 |
| 2 | 234233883 | | Direct bilirubin | 0.66 | 6.54E-09 |
| 2 | 234415488 | | Indirect bilirubin | 0.88 | 6.54E-09 |
| 17 | 38157021 | | Neutrophil count | -0.45 | 6.68E-09 |
| 2 | 234225681 | | Direct bilirubin | 0.61 | 6.70E-09 |
| 20 | 57558382 | | Platelet distribution width | 0.08 | 6.81E-09 |
| 7 | 140623286 | | Gestation | -1.88 | 6.82E-09 |
| 6 | 160693727 | | Lipoprotein | -40.31 | 6.83E-09 |
| 2 | 234334512 | | Indirect bilirubin | -0.68 | 6.87E-09 |
| 2 | 234455344 | | Total bilirubin | 1.35 | 6.89E-09 |
| 2 | 234466068 | | Indirect bilirubin | 0.91 | 7.03E-09 |
| 7 | 140529133 | | Gestation | -1.88 | 7.08E-09 |
| 2 | 46582891 | | Altitude | -106.96 | 7.09E-09 |
| 2 | 234460046 | | Indirect bilirubin | 0.88 | 7.09E-09 |
| 2 | 234483706 | | Indirect bilirubin | 0.90 | 7.10E-09 |
| 2 | 234637192 | | DBIL/TBIL ratio | -0.01 | 7.25E-09 |
| 2 | 233261411 | | Alkaline phosphatase | -22.91 | 7.28E-09 |
| 2 | 234469013 | | Total bilirubin | 1.35 | 7.29E-09 |
| 2 | 234469233 | | Indirect bilirubin | 0.86 | 7.38E-09 |
| 2 | 234500532 | | Total bilirubin | 1.37 | 7.47E-09 |
| 17 | 38189055 | | White blood cell count | -0.48 | 7.48E-09 |
| 2 | 234451882 | | Total bilirubin | 1.34 | 7.51E-09 |
| 2 | 46629693 | | Altitude | -99.49 | 7.54E-09 |
| 6 | 160693736 | | Lipoprotein | -40.21 | 7.54E-09 |
| 17 | 38215948 | | White blood cell count | -0.47 | 7.54E-09 |
| 11 | 125818297 | | Hemoglobin | -4.30 | 7.56E-09 |
| 2 | 46597581 | | Altitude | -107.49 | 7.61E-09 |
| 2 | 234420787 | | Total bilirubin | 1.40 | 7.62E-09 |
| 2 | 234497246 | | Indirect bilirubin | 0.92 | 7.84E-09 |
| 2 | 169870855 | | Gamma-glutamyl transpeptidase | 2.17 | 7.85E-09 |
| 7 | 140519239 | | Gestation | -1.91 | 7.92E-09 |
| 2 | 234508768 | | Indirect bilirubin | 0.94 | 8.09E-09 |
| 2 | 234506223 | | Direct bilirubin | 0.38 | 8.12E-09 |
| 2 | 234511864 | | Direct bilirubin | 0.38 | 8.14E-09 |
| 2 | 46597827 | | Altitude | -98.61 | 8.14E-09 |
| 1 | 11979909 | | Thyroid stimulating hormone | -0.46 | 8.15E-09 |
| 17 | 38215314 | | White blood cell count | -0.47 | 8.21E-09 |
| 1 | 11968273 | | Thyroid stimulating hormone | -0.44 | 8.21E-09 |
| 2 | 234255145 | | Indirect bilirubin | -0.57 | 8.31E-09 |
| 2 | 234508768 | | Direct bilirubin | 0.37 | 8.32E-09 |
| 2 | 234210329 | | Total bilirubin | 0.97 | 8.64E-09 |
| 2 | 234142691 | | Total bilirubin | 1.76 | 8.67E-09 |
| 2 | 234687371 | | Direct bilirubin | 0.63 | 8.72E-09 |
| 2 | 234419534 | | Indirect bilirubin | 0.88 | 8.80E-09 |
| 2 | 234280388 | | Indirect bilirubin | -0.64 | 8.92E-09 |
| 2 | 234241662 | | Total bilirubin | 1.70 | 9.07E-09 |
| 2 | 234253597 | | Direct bilirubin | -0.32 | 9.13E-09 |
| 2 | 234650193 | | DBIL/TBIL ratio | -0.01 | 9.16E-09 |
| 2 | 234513372 | | Indirect bilirubin | 0.95 | 9.16E-09 |
| 2 | 234234538 | | Direct bilirubin | 0.62 | 9.18E-09 |
| 2 | 234408145 | | Indirect bilirubin | 0.86 | 9.19E-09 |
| 2 | 234416178 | | Direct bilirubin | 0.52 | 9.21E-09 |
| 2 | 234527397 | | DBIL/TBIL ratio | 0.01 | 9.23E-09 |
| 2 | 234334512 | | Total bilirubin | -1.02 | 9.35E-09 |
| 2 | 234415925 | | Total bilirubin | 1.32 | 9.40E-09 |
| 2 | 234502121 | | Total bilirubin | 1.30 | 9.42E-09 |
| 2 | 234210329 | | Indirect bilirubin | 0.81 | 9.54E-09 |
| 2 | 234381780 | | Total bilirubin | 1.33 | 9.55E-09 |
| 2 | 169868827 | | Gamma-glutamyl transpeptidase | 2.20 | 9.60E-09 |
| 6 | 160508142 | | Lipoprotein | 59.76 | 9.66E-09 |
| 2 | 234451044 | | Indirect bilirubin | 0.88 | 9.66E-09 |
| 2 | 234469208 | | Indirect bilirubin | 0.86 | 9.80E-09 |
| 2 | 234204672 | | Indirect bilirubin | -0.54 | 9.93E-09 |
| 2 | 234471832 | | Direct bilirubin | 0.50 | 1.02E-08 |
| 2 | 234491330 | | Indirect bilirubin | 0.90 | 1.05E-08 |
| 2 | 234583490 | | Direct bilirubin | 0.29 | 1.05E-08 |
| 6 | 160695320 | | Lipoprotein | -40.16 | 1.08E-08 |
| 2 | 234702459 | | Total bilirubin | -0.87 | 1.08E-08 |
| 4 | 12415441 | | Hematocrit | 0.95 | 1.09E-08 |
| 2 | 234418873 | | Total bilirubin | 1.36 | 1.10E-08 |
| 2 | 234233593 | | Direct bilirubin | 0.62 | 1.11E-08 |
| 1 | 11973842 | | Phosphorus | 1.44 | 1.11E-08 |
| 11 | 125800619 | | Hemoglobin | -4.26 | 1.12E-08 |
| 5 | 176821449 | | Activated fraction thromboplastin time | -0.84 | 1.13E-08 |
| 1 | 11960056 | | Phosphorus | 1.34 | 1.13E-08 |
| 2 | 234491725 | | Total bilirubin | 1.32 | 1.14E-08 |
| 17 | 38169714 | | Neutrophil count | -0.44 | 1.15E-08 |
| 11 | 116601945 | | Triglyceride | 0.09 | 1.15E-08 |
| 2 | 234616357 | | Indirect bilirubin | 0.56 | 1.17E-08 |
| 20 | 57576174 | | Platelet distribution width | 0.09 | 1.17E-08 |
| 2 | 234638690 | | DBIL/TBIL ratio | -0.01 | 1.19E-08 |
| 2 | 234497823 | | Direct bilirubin | 0.50 | 1.20E-08 |
| 2 | 234612690 | | DBIL/TBIL ratio | -0.01 | 1.21E-08 |
| 2 | 234687371 | | Total bilirubin | 1.69 | 1.21E-08 |
| 20 | 57578508 | | Platelet distribution width | 0.08 | 1.22E-08 |
| 2 | 234475297 | | Indirect bilirubin | 0.85 | 1.22E-08 |
| 1 | 11937404 | | Phosphorus | 1.39 | 1.24E-08 |
| 2 | 234676872 | | Indirect bilirubin | 1.08 | 1.24E-08 |
| 2 | 234227118 | | Direct bilirubin | 0.60 | 1.25E-08 |
| 2 | 46589179 | | Altitude | -106.43 | 1.25E-08 |
| 2 | 234493876 | | Total bilirubin | 1.32 | 1.26E-08 |
| 2 | 234462748 | | Indirect bilirubin | 0.86 | 1.27E-08 |
| 20 | 57576180 | | Platelet distribution width | 0.08 | 1.27E-08 |
| 2 | 234637803 | | DBIL/TBIL ratio | -0.01 | 1.28E-08 |
| 2 | 234647478 | | DBIL/TBIL ratio | -0.01 | 1.28E-08 |
| 2 | 234610477 | | Total bilirubin | 1.19 | 1.30E-08 |
| 2 | 46753277 | | Red blood cell count | 0.10 | 1.30E-08 |
| 1 | 12002164 | | Thyroid stimulating hormone | 0.41 | 1.30E-08 |
| 2 | 234417312 | | Total bilirubin | 1.31 | 1.32E-08 |
| 2 | 234427312 | | Total bilirubin | 1.30 | 1.32E-08 |
| 2 | 234402375 | | Total bilirubin | 1.31 | 1.32E-08 |
| 2 | 234248110 | | Indirect bilirubin | -0.53 | 1.33E-08 |
| 2 | 234415488 | | Total bilirubin | 1.32 | 1.34E-08 |
| 2 | 234406021 | | Total bilirubin | 1.35 | 1.35E-08 |
| 2 | 234224127 | | Total bilirubin | 1.86 | 1.36E-08 |
| 2 | 234461161 | | Indirect bilirubin | 0.88 | 1.36E-08 |
| 17 | 38168227 | | Neutrophil count | -0.43 | 1.36E-08 |
| 2 | 234491462 | | Indirect bilirubin | 0.87 | 1.38E-08 |
| 2 | 234258101 | | Direct bilirubin | -0.31 | 1.39E-08 |
| 2 | 234298826 | | Direct bilirubin | -0.36 | 1.41E-08 |
| 2 | 234492961 | | Indirect bilirubin | 0.92 | 1.41E-08 |
| 2 | 234483706 | | Total bilirubin | 1.34 | 1.42E-08 |
| 2 | 234457312 | | Total bilirubin | 1.30 | 1.42E-08 |
| 2 | 234465134 | | Indirect bilirubin | 0.87 | 1.42E-08 |
| 2 | 234246137 | | Direct bilirubin | -0.30 | 1.43E-08 |
| 2 | 234418405 | | Total bilirubin | 1.32 | 1.44E-08 |
| 17 | 38205793 | | White blood cell count | -0.47 | 1.44E-08 |
| 2 | 234676872 | | Direct bilirubin | 0.60 | 1.45E-08 |
| 2 | 234459592 | | Indirect bilirubin | 0.92 | 1.46E-08 |
| 20 | 57558481 | | Platelet distribution width | 0.09 | 1.47E-08 |
| 7 | 140520733 | | Gestation | -1.83 | 1.47E-08 |
| 2 | 234463892 | | Indirect bilirubin | 0.87 | 1.48E-08 |
| 2 | 234332439 | | Direct bilirubin | 0.48 | 1.48E-08 |
| 2 | 234233593 | | Indirect bilirubin | 1.06 | 1.49E-08 |
| 2 | 234421754 | | Total bilirubin | 1.30 | 1.49E-08 |
| 1 | 11975166 | | Thyroid stimulating hormone | -0.45 | 1.49E-08 |
| 11 | 125813286 | | Hemoglobin | -4.19 | 1.50E-08 |
| 2 | 234405690 | | Indirect bilirubin | 0.87 | 1.51E-08 |
| 6 | 161121715 | | Lipoprotein | 54.94 | 1.52E-08 |
| 2 | 234617365 | | Indirect bilirubin | 0.55 | 1.53E-08 |
| 2 | 234610477 | | Indirect bilirubin | 1.16 | 1.54E-08 |
| 2 | 234163683 | | Total bilirubin | -0.85 | 1.56E-08 |
| 2 | 234412079 | | Total bilirubin | 1.33 | 1.56E-08 |
| 2 | 234466068 | | Total bilirubin | 1.35 | 1.57E-08 |
| 2 | 234665659 | | DBIL/TBIL ratio | -0.01 | 1.57E-08 |
| 2 | 234673462 | | DBIL/TBIL ratio | -0.01 | 1.58E-08 |
| 20 | 57560777 | | Platelet distribution width | 0.08 | 1.58E-08 |
| 2 | 234668870 | | Total bilirubin | 1.70 | 1.59E-08 |
| 2 | 234420754 | | Total bilirubin | 1.38 | 1.59E-08 |
| 2 | 234668870 | | Direct bilirubin | 0.63 | 1.60E-08 |
| 1 | 156868848 | | Platelets | -11.68 | 1.62E-08 |
| 2 | 234322805 | | Indirect bilirubin | 0.88 | 1.62E-08 |
| 2 | 234417428 | | Total bilirubin | 1.31 | 1.62E-08 |
| 2 | 234483673 | | Total bilirubin | 1.31 | 1.64E-08 |
| 2 | 234235454 | | Direct bilirubin | 0.61 | 1.64E-08 |
| 2 | 234247924 | | Total bilirubin | -0.81 | 1.64E-08 |
| 2 | 234466068 | | Direct bilirubin | 0.50 | 1.64E-08 |
| 2 | 234204672 | | Direct bilirubin | -0.30 | 1.65E-08 |
| 2 | 234154614 | | Total bilirubin | 1.77 | 1.65E-08 |
| 10 | 31476778 | | Corrected visual acuity of the right eye | 0.87 | 1.66E-08 |
| 2 | 234481410 | | Indirect bilirubin | 0.86 | 1.67E-08 |
| 2 | 234494513 | | Direct bilirubin | 0.49 | 1.68E-08 |
| 2 | 234410508 | | Indirect bilirubin | 0.88 | 1.68E-08 |
| 2 | 234506223 | | Total bilirubin | 1.43 | 1.68E-08 |
| 2 | 234411029 | | Total bilirubin | 1.30 | 1.68E-08 |
| 20 | 57564070 | | Platelet distribution width | 0.08 | 1.69E-08 |
| 2 | 234415702 | | Indirect bilirubin | 0.86 | 1.69E-08 |
| 9 | 128798584 | | Mean corpuscular hemoglobin concentration | 1.90 | 1.69E-08 |
| 2 | 234508692 | | Direct bilirubin | 0.37 | 1.69E-08 |
| 2 | 234278549 | | Direct bilirubin | -0.36 | 1.70E-08 |
| 20 | 57559842 | | Platelet distribution width | 0.08 | 1.71E-08 |
| 2 | 234419637 | | Indirect bilirubin | 0.86 | 1.72E-08 |
| 2 | 234472396 | | Total bilirubin | 1.29 | 1.73E-08 |
| 2 | 234445799 | | Total bilirubin | 1.28 | 1.73E-08 |
| 2 | 234430931 | | Total bilirubin | 1.31 | 1.73E-08 |
| 2 | 234617365 | | Direct bilirubin | 0.42 | 1.73E-08 |
| 2 | 234475235 | | Total bilirubin | 1.32 | 1.74E-08 |
| 2 | 234168042 | | Direct bilirubin | 0.67 | 1.74E-08 |
| 7 | 140495542 | | Gestation | -1.85 | 1.74E-08 |
| 7 | 140445037 | | Gestation | -1.86 | 1.75E-08 |
| 7 | 140475367 | | Gestation | -1.85 | 1.75E-08 |
| 20 | 57567543 | | Platelet distribution width | 0.08 | 1.77E-08 |
| 2 | 234405739 | | Indirect bilirubin | 0.84 | 1.77E-08 |
| 2 | 234461161 | | Total bilirubin | 1.33 | 1.79E-08 |
| 2 | 169852842 | | Gamma-glutamyl transpeptidase | 2.08 | 1.79E-08 |
| 2 | 234605835 | | Direct bilirubin | 0.42 | 1.79E-08 |
| 2 | 234233883 | | Total bilirubin | 1.74 | 1.80E-08 |
| 2 | 234246458 | | Total bilirubin | -0.83 | 1.81E-08 |
| 2 | 234418427 | | Direct bilirubin | 0.48 | 1.82E-08 |
| 2 | 234752638 | | Direct bilirubin | 0.39 | 1.83E-08 |
| 2 | 234456486 | | Indirect bilirubin | 0.87 | 1.86E-08 |
| 11 | 116583439 | | Triglyceride | 0.09 | 1.86E-08 |
| 2 | 234465134 | | Total bilirubin | 1.32 | 1.89E-08 |
| 2 | 233288749 | | Alkaline phosphatase | -13.00 | 1.89E-08 |
| 2 | 234641908 | | DBIL/TBIL ratio | -0.01 | 1.91E-08 |
| 2 | 234424293 | | Indirect bilirubin | 0.84 | 1.92E-08 |
| 2 | 234458612 | | Indirect bilirubin | 0.86 | 1.93E-08 |
| 2 | 234411435 | | Indirect bilirubin | 0.84 | 1.94E-08 |
| 2 | 234460046 | | Total bilirubin | 1.30 | 1.95E-08 |
| 2 | 233261358 | | Alkaline phosphatase | -22.98 | 1.95E-08 |
| 11 | 116603134 | | Triglyceride | 0.09 | 1.97E-08 |
| 2 | 234635467 | | DBIL/TBIL ratio | -0.01 | 1.97E-08 |
| 2 | 234415488 | | Direct bilirubin | 0.48 | 1.98E-08 |
| 2 | 234248110 | | Direct bilirubin | -0.30 | 2.01E-08 |
| 2 | 234256849 | | Direct bilirubin | -0.31 | 2.01E-08 |
| 2 | 234491077 | | Total bilirubin | 1.29 | 2.02E-08 |
| 2 | 234208045 | | Direct bilirubin | 0.67 | 2.02E-08 |
| 2 | 46609957 | | Altitude | -101.27 | 2.03E-08 |
| 11 | 116589127 | | Triglyceride | 0.09 | 2.03E-08 |
| 2 | 234499326 | | Total bilirubin | 1.29 | 2.04E-08 |
| 1 | 236879793 | | Alkaline phosphatase | 16.18 | 2.05E-08 |
| 1 | 11950391 | | Phosphorus | 1.59 | 2.05E-08 |
| 8 | 53915883 | | Coefficient of variation of red blood cell | 0.33 | 2.06E-08 |
| 2 | 234464134 | | Direct bilirubin | 0.51 | 2.09E-08 |
| 2 | 234402411 | | Total bilirubin | 1.28 | 2.09E-08 |
| 2 | 234581920 | | Direct bilirubin | 0.28 | 2.09E-08 |
| 2 | 234245384 | | Direct bilirubin | 0.68 | 2.13E-08 |
| 2 | 233298315 | | Alkaline phosphatase | -13.36 | 2.15E-08 |
| 2 | 234497246 | | Total bilirubin | 1.37 | 2.16E-08 |
| 2 | 234252910 | | Indirect bilirubin | -0.56 | 2.18E-08 |
| 2 | 234298826 | | Indirect bilirubin | -0.55 | 2.18E-08 |
| 7 | 140556557 | | Gestation | -1.78 | 2.19E-08 |
| 2 | 234247924 | | Direct bilirubin | -0.30 | 2.20E-08 |
| 2 | 234412530 | | Total bilirubin | 1.29 | 2.20E-08 |
| 2 | 234171459 | | Direct bilirubin | 0.66 | 2.22E-08 |
| 1 | 156869630 | | Platelets | -11.59 | 2.23E-08 |
| 2 | 46614202 | | Altitude | -89.15 | 2.24E-08 |
| 2 | 234319220 | | Indirect bilirubin | 0.86 | 2.25E-08 |
| 2 | 234464969 | | Indirect bilirubin | 0.84 | 2.26E-08 |
| 1 | 156867327 | | Platelets | -11.49 | 2.27E-08 |
| 2 | 234644421 | | DBIL/TBIL ratio | -0.01 | 2.29E-08 |
| 17 | 38157920 | | Neutrophil count | -0.43 | 2.30E-08 |
| 2 | 234164492 | | Direct bilirubin | 0.66 | 2.30E-08 |
| 2 | 234420787 | | Direct bilirubin | 0.50 | 2.30E-08 |
| 2 | 46614235 | | Altitude | -89.30 | 2.31E-08 |
| 2 | 234253597 | | Total bilirubin | -0.84 | 2.32E-08 |
| 2 | 234694177 | | Indirect bilirubin | -0.73 | 2.33E-08 |
| 2 | 234468042 | | Total bilirubin | 1.29 | 2.34E-08 |
| 2 | 234193274 | | Indirect bilirubin | 0.55 | 2.36E-08 |
| 2 | 234539111 | | Indirect bilirubin | 0.59 | 2.36E-08 |
| 2 | 46583593 | | Altitude | -103.10 | 2.38E-08 |
| 2 | 234417260 | | Indirect bilirubin | 0.82 | 2.40E-08 |
| 20 | 57564448 | | Platelet distribution width | 0.08 | 2.41E-08 |
| 20 | 57567730 | | Platelet distribution width | 0.08 | 2.42E-08 |
| 2 | 234467708 | | Indirect bilirubin | 0.86 | 2.42E-08 |
| 2 | 234483489 | | Total bilirubin | 1.29 | 2.42E-08 |
| 2 | 46688833 | | Red blood cell count | 0.10 | 2.43E-08 |
| 11 | 125795702 | | Hemoglobin | -4.14 | 2.44E-08 |
| 2 | 234334512 | | Direct bilirubin | -0.37 | 2.44E-08 |
| 2 | 234234856 | | Indirect bilirubin | 1.03 | 2.44E-08 |
| 2 | 234255145 | | Total bilirubin | -0.81 | 2.45E-08 |
| 2 | 233289781 | | Alkaline phosphatase | -12.94 | 2.45E-08 |
| 17 | 38169273 | | Neutrophil count | -0.43 | 2.47E-08 |
| 2 | 234154936 | | Total bilirubin | 1.76 | 2.50E-08 |
| 1 | 11979556 | | Thyroid stimulating hormone | -0.44 | 2.51E-08 |
| 2 | 234252910 | | Direct bilirubin | -0.31 | 2.53E-08 |
| 11 | 116589652 | | Triglyceride | 0.09 | 2.54E-08 |
| 2 | 234411118 | | Indirect bilirubin | 0.85 | 2.54E-08 |
| 2 | 169845026 | | Gamma-glutamyl transpeptidase | 2.04 | 2.55E-08 |
| 1 | 11983206 | | Thyroid stimulating hormone | -0.45 | 2.56E-08 |
| 2 | 234252910 | | Total bilirubin | -0.84 | 2.57E-08 |
| 2 | 234508692 | | Indirect bilirubin | 0.90 | 2.57E-08 |
| 11 | 116585533 | | Triglyceride | 0.09 | 2.58E-08 |
| 2 | 234455213 | | Total bilirubin | 1.27 | 2.59E-08 |
| 2 | 234381780 | | Direct bilirubin | 0.48 | 2.60E-08 |
| 20 | 57577505 | | Platelet distribution width | 0.08 | 2.60E-08 |
| 1 | 236855635 | | Alkaline phosphatase | 16.18 | 2.62E-08 |
| 2 | 46610680 | | Altitude | -100.44 | 2.63E-08 |
| 2 | 234652740 | | DBIL/TBIL ratio | 0.02 | 2.63E-08 |
| 2 | 234516799 | | Total bilirubin | 1.39 | 2.63E-08 |
| 2 | 46614463 | | Altitude | -88.30 | 2.63E-08 |
| 17 | 38161823 | | Neutrophil count | -0.43 | 2.63E-08 |
| 7 | 140592257 | | Gestation | -1.79 | 2.64E-08 |
| 2 | 234452015 | | Total bilirubin | 1.27 | 2.65E-08 |
| 2 | 234163683 | | Direct bilirubin | -0.31 | 2.66E-08 |
| 2 | 234637707 | | DBIL/TBIL ratio | -0.01 | 2.66E-08 |
| 20 | 57578852 | | Platelet distribution width | 0.08 | 2.66E-08 |
| 2 | 234234856 | | Direct bilirubin | 0.60 | 2.67E-08 |
| 2 | 234257452 | | Direct bilirubin | -0.31 | 2.67E-08 |
| 2 | 233266881 | | Alkaline phosphatase | -21.73 | 2.70E-08 |
| 2 | 46626352 | | Altitude | -96.52 | 2.71E-08 |
| 2 | 234258101 | | Total bilirubin | -0.82 | 2.72E-08 |
| 2 | 234453351 | | Indirect bilirubin | 0.86 | 2.75E-08 |
| 2 | 233295063 | | Alkaline phosphatase | -12.93 | 2.76E-08 |
| 2 | 233295064 | | Alkaline phosphatase | -12.93 | 2.76E-08 |
| 2 | 233295066 | | Alkaline phosphatase | -12.93 | 2.76E-08 |
| 17 | 38215695 | | Neutrophil count | -0.42 | 2.79E-08 |
| 2 | 234702459 | | Direct bilirubin | -0.31 | 2.79E-08 |
| 10 | 31453054 | | Corrected visual acuity of the right eye | 0.69 | 2.80E-08 |
| 2 | 234637853 | | DBIL/TBIL ratio | -0.01 | 2.81E-08 |
| 1 | 11937404 | | Thyroid stimulating hormone | -0.40 | 2.82E-08 |
| 2 | 234635241 | | DBIL/TBIL ratio | -0.01 | 2.85E-08 |
| 2 | 234650604 | | DBIL/TBIL ratio | -0.01 | 2.85E-08 |
| 7 | 140540041 | | Gestation | -1.76 | 2.86E-08 |
| 2 | 234313808 | | Indirect bilirubin | 0.83 | 2.88E-08 |
| 2 | 234313809 | | Indirect bilirubin | 0.83 | 2.88E-08 |
| 2 | 234469233 | | Total bilirubin | 1.27 | 2.89E-08 |
| 2 | 234513372 | | Direct bilirubin | 0.36 | 2.90E-08 |
| 20 | 57578614 | | Platelet distribution width | 0.08 | 2.91E-08 |
| 2 | 234384293 | | Total bilirubin | 1.26 | 2.91E-08 |
| 2 | 234246264 | | Direct bilirubin | -0.30 | 2.91E-08 |
| 2 | 234489656 | | Direct bilirubin | 0.49 | 2.92E-08 |
| 1 | 236866836 | | Alkaline phosphatase | 16.72 | 2.92E-08 |
| 10 | 114692056 | | Free thyroxine | -41.60 | 2.92E-08 |
| 2 | 234265921 | | Direct bilirubin | -0.35 | 2.93E-08 |
| 2 | 234401581 | | Total bilirubin | 1.25 | 2.93E-08 |
| 2 | 234233019 | | Total bilirubin | 1.50 | 2.93E-08 |
| 2 | 234227118 | | Indirect bilirubin | 1.04 | 2.95E-08 |
| 2 | 234193274 | | Total bilirubin | 0.81 | 2.96E-08 |
| 2 | 234147256 | | Direct bilirubin | 0.65 | 2.96E-08 |
| 2 | 234610477 | | Direct bilirubin | 0.64 | 2.97E-08 |
| 2 | 234602641 | | Direct bilirubin | 0.28 | 2.98E-08 |
| 1 | 11949970 | | Thyroid stimulating hormone | -0.46 | 3.01E-08 |
| 2 | 234319220 | | Total bilirubin | 1.31 | 3.02E-08 |
| 2 | 46630395 | | Altitude | -88.78 | 3.02E-08 |
| 2 | 234265921 | | Indirect bilirubin | -0.61 | 3.03E-08 |
| 2 | 234265921 | | Total bilirubin | -0.93 | 3.03E-08 |
| 2 | 233257043 | | Alkaline phosphatase | -21.88 | 3.03E-08 |
| 2 | 46622245 | | Altitude | -88.40 | 3.08E-08 |
| 2 | 46582382 | | Altitude | -101.57 | 3.09E-08 |
| 2 | 21473640 | | Low density lipoprotein | -0.18 | 3.11E-08 |
| 2 | 234514763 | | Total bilirubin | 1.39 | 3.15E-08 |
| 2 | 234278549 | | Indirect bilirubin | -0.54 | 3.18E-08 |
| 2 | 234322805 | | Total bilirubin | 1.31 | 3.19E-08 |
| 2 | 234469725 | | Indirect bilirubin | 0.90 | 3.19E-08 |
| 2 | 234234922 | | Direct bilirubin | 0.59 | 3.21E-08 |
| 2 | 234502121 | | Direct bilirubin | 0.34 | 3.26E-08 |
| 2 | 46581544 | | Altitude | -103.35 | 3.29E-08 |
| 9 | 107661561 | | High density lipoprotein | -0.04 | 3.30E-08 |
| 2 | 234256849 | | Total bilirubin | -0.82 | 3.30E-08 |
| 2 | 234465572 | | Indirect bilirubin | 0.83 | 3.33E-08 |
| 2 | 234408547 | | Total bilirubin | 1.25 | 3.34E-08 |
| 7 | 140604660 | | Gestation | -1.76 | 3.34E-08 |
| 2 | 46752876 | | Red blood cell count | 0.10 | 3.35E-08 |
| 2 | 234439664 | | Indirect bilirubin | 0.86 | 3.38E-08 |
| 2 | 234400404 | | Total bilirubin | 1.28 | 3.38E-08 |
| 2 | 46605659 | | Altitude | -100.26 | 3.39E-08 |
| 2 | 234634916 | | DBIL/TBIL ratio | -0.01 | 3.40E-08 |
| 2 | 234469208 | | Total bilirubin | 1.27 | 3.40E-08 |
| 1 | 37620309 | | Standard deviation of variation of red blood cell | 0.69 | 3.41E-08 |
| 2 | 234381817 | | DBIL/TBIL ratio | 0.01 | 3.44E-08 |
| 2 | 46624372 | | Altitude | -87.26 | 3.44E-08 |
| 2 | 234567051 | | Total bilirubin | 1.29 | 3.47E-08 |
| 20 | 57575281 | | Platelet distribution width | 0.07 | 3.48E-08 |
| 2 | 234418209 | | Total bilirubin | 1.25 | 3.50E-08 |
| 2 | 234605835 | | Indirect bilirubin | 0.54 | 3.50E-08 |
| 2 | 46584573 | | Altitude | -103.10 | 3.51E-08 |
| 2 | 46590384 | | Altitude | -103.42 | 3.52E-08 |
| 10 | 31469246 | | Corrected visual acuity of the right eye | 0.71 | 3.52E-08 |
| 2 | 234280388 | | Total bilirubin | -0.93 | 3.53E-08 |
| 2 | 169856433 | | Gamma-glutamyl transpeptidase | 2.02 | 3.55E-08 |
| 20 | 57579950 | | Platelet distribution width | 0.08 | 3.55E-08 |
| 2 | 234193663 | | Direct bilirubin | 0.64 | 3.56E-08 |
| 2 | 234460385 | | Direct bilirubin | 0.48 | 3.59E-08 |
| 2 | 234462748 | | Total bilirubin | 1.27 | 3.60E-08 |
| 2 | 234216917 | | Indirect bilirubin | 1.04 | 3.61E-08 |
| 2 | 234469725 | | Total bilirubin | 1.36 | 3.61E-08 |
| 9 | 89843699 | | Potassium | -0.05 | 3.62E-08 |
| 2 | 234517082 | | Indirect bilirubin | 0.93 | 3.62E-08 |
| 2 | 234491647 | | Direct bilirubin | 0.49 | 3.64E-08 |
| 2 | 234461161 | | Direct bilirubin | 0.48 | 3.64E-08 |
| 2 | 234154936 | | Direct bilirubin | 0.66 | 3.65E-08 |
| 2 | 234639971 | | Direct bilirubin | 0.36 | 3.72E-08 |
| 2 | 137065275 | | Total bile acid | 0.57 | 3.73E-08 |
| 1 | 12018290 | | Thyroid stimulating hormone | 0.37 | 3.75E-08 |
| 2 | 234257452 | | Total bilirubin | -0.82 | 3.76E-08 |
| 9 | 89820921 | | Potassium | -0.05 | 3.76E-08 |
| 2 | 234506223 | | Total bilirubin | 0.92 | 3.77E-08 |
| 2 | 234467645 | | Indirect bilirubin | 0.87 | 3.79E-08 |
| 2 | 234185759 | | Direct bilirubin | 0.64 | 3.79E-08 |
| 2 | 234415835 | | Indirect bilirubin | 0.84 | 3.81E-08 |
| 2 | 234620272 | | DBIL/TBIL ratio | -0.01 | 3.82E-08 |
| 2 | 169879887 | | Gamma-glutamyl transpeptidase | 2.16 | 3.84E-08 |
| 2 | 234517082 | | Total bilirubin | 1.42 | 3.86E-08 |
| 2 | 234419534 | | Total bilirubin | 1.29 | 3.87E-08 |
| 2 | 169850718 | | Gamma-glutamyl transpeptidase | 2.01 | 3.89E-08 |
| 7 | 140544307 | | Gestation | -1.80 | 3.93E-08 |
| 1 | 11968382 | | Phosphorus | 1.35 | 3.94E-08 |
| 2 | 234525824 | | Indirect bilirubin | 0.93 | 3.94E-08 |
| 2 | 234278549 | | DBIL/TBIL ratio | 0.01 | 3.96E-08 |
| 2 | 234246994 | | Direct bilirubin | -0.30 | 3.96E-08 |
| 2 | 234516799 | | Indirect bilirubin | 0.90 | 3.98E-08 |
| 6 | 161115245 | | Lipoprotein | 55.48 | 3.99E-08 |
| 5 | 104943393 | | Triglyceride | -0.07 | 4.00E-08 |
| 2 | 234525824 | | Total bilirubin | 1.41 | 4.01E-08 |
| 15 | 52526677 | | Alkaline phosphatase | 10.86 | 4.03E-08 |
| 2 | 234319220 | | Direct bilirubin | 0.48 | 4.05E-08 |
| 9 | 89820895 | | Potassium | -0.05 | 4.08E-08 |
| 2 | 234516799 | | Direct bilirubin | 0.36 | 4.10E-08 |
| 2 | 234401268 | | Indirect bilirubin | 0.83 | 4.10E-08 |
| 2 | 234539111 | | Total bilirubin | 1.32 | 4.10E-08 |
| 9 | 128798476 | | Mean corpuscular hemoglobin concentration | 1.87 | 4.12E-08 |
| 2 | 234420754 | | Direct bilirubin | 0.50 | 4.13E-08 |
| 2 | 234584324 | | Direct bilirubin | 0.40 | 4.16E-08 |
| 9 | 114887801 | | Glutamate pyruvate transaminase | 6.43 | 4.16E-08 |
| 2 | 169843995 | | Gamma-glutamyl transpeptidase | 2.01 | 4.17E-08 |
| 2 | 46701480 | | Red blood cell count | 0.10 | 4.19E-08 |
| 2 | 234422821 | | Direct bilirubin | 0.47 | 4.19E-08 |
| 2 | 234458949 | | Indirect bilirubin | 0.83 | 4.22E-08 |
| 2 | 234463892 | | Total bilirubin | 1.29 | 4.25E-08 |
| 2 | 234518958 | | Indirect bilirubin | 0.93 | 4.29E-08 |
| 2 | 234517722 | | Total bilirubin | 1.39 | 4.31E-08 |
| 2 | 234409069 | | Indirect bilirubin | 0.83 | 4.35E-08 |
| 17 | 38213443 | | Neutrophil count | -0.42 | 4.35E-08 |
| 1 | 11995409 | | Thyroid stimulating hormone | -0.49 | 4.36E-08 |
| 2 | 234322805 | | Direct bilirubin | 0.48 | 4.36E-08 |
| 2 | 234455344 | | Direct bilirubin | 0.47 | 4.38E-08 |
| 2 | 234233066 | | Direct bilirubin | 0.60 | 4.40E-08 |
| 2 | 234493048 | | Direct bilirubin | 0.47 | 4.41E-08 |
| 2 | 234506223 | | Indirect bilirubin | 0.91 | 4.41E-08 |
| 2 | 234475297 | | Total bilirubin | 1.25 | 4.42E-08 |
| 7 | 140589660 | | Gestation | -1.74 | 4.42E-08 |
| 2 | 234153848 | | Total bilirubin | -0.83 | 4.43E-08 |
| 1 | 68145114 | | Coefficient of variation of red blood cell | -0.27 | 4.44E-08 |
| 2 | 234256849 | | Indirect bilirubin | -0.53 | 4.45E-08 |
| 2 | 234751581 | | Direct bilirubin | 0.37 | 4.45E-08 |
| 7 | 140500135 | | Gestation | -1.75 | 4.47E-08 |
| 2 | 234405739 | | Total bilirubin | 1.25 | 4.48E-08 |
| 2 | 234153848 | | Direct bilirubin | -0.31 | 4.50E-08 |
| 6 | 160999191 | | Lipoprotein | 37.56 | 4.54E-08 |
| 2 | 234449316 | | Direct bilirubin | 0.42 | 4.55E-08 |
| 2 | 234687371 | | Indirect bilirubin | 1.06 | 4.56E-08 |
| 11 | 116588909 | | Triglyceride | 0.09 | 4.56E-08 |
| 1 | 156869237 | | Platelets | -11.25 | 4.59E-08 |
| 9 | 107571375 | | High density lipoprotein | -0.04 | 4.62E-08 |
| 2 | 234439664 | | Total bilirubin | 1.30 | 4.62E-08 |
| 16 | 7229376 | | PH value | -0.02 | 4.64E-08 |
| 2 | 234401734 | | Indirect bilirubin | 0.82 | 4.66E-08 |
| 1 | 11968395 | | Phosphorus | 1.33 | 4.67E-08 |
| 7 | 140466450 | | Gestation | -1.77 | 4.67E-08 |
| 2 | 169847412 | | Gamma-glutamyl transpeptidase | 2.00 | 4.68E-08 |
| 2 | 234492961 | | Total bilirubin | 1.31 | 4.70E-08 |
| 7 | 140609213 | | Gestation | -1.73 | 4.71E-08 |
| 2 | 234246458 | | Indirect bilirubin | -0.53 | 4.73E-08 |
| 2 | 234247924 | | Indirect bilirubin | -0.52 | 4.77E-08 |
| 2 | 234619937 | | Indirect bilirubin | 0.53 | 4.78E-08 |
| 1 | 12076347 | | Phosphorus | 1.64 | 4.80E-08 |
| 2 | 234622429 | | DBIL/TBIL ratio | -0.01 | 4.81E-08 |
| 2 | 234253597 | | Indirect bilirubin | -0.54 | 4.82E-08 |
| 2 | 234579892 | | Direct bilirubin | 0.28 | 4.85E-08 |
| 2 | 234539111 | | Indirect bilirubin | 0.86 | 4.86E-08 |
| 7 | 140427699 | | Gestation | -1.77 | 4.87E-08 |
| 2 | 46630861 | | Altitude | -85.56 | 4.89E-08 |
| 7 | 140459344 | | Gestation | -1.77 | 4.89E-08 |
| 1 | 12016772 | | Thyroid stimulating hormone | 0.37 | 4.91E-08 |
| 2 | 234153848 | | Direct bilirubin | -0.26 | 4.91E-08 |
| 1 | 242920580 | | Hemoglobin | - | 4.96E-08 |
| 6 | 160915695 | | Lipoprotein | -61.82 | 4.96E-08 |
| 17 | 38169095 | | Neutrophil count | -0.42 | 4.98E-08 |
| 7 | 140449973 | | Gestation | -1.80 | 4.98E-08 |
| 20 | 57557579 | | Platelet distribution width | 0.08 | 4.98E-08 |
| 2 | 234518958 | | Total bilirubin | 1.42 | 4.99E-08 |
