# Supplementary material for: HiLand Resource: A Comprehensive Database of Highland Human Populations
Source: Genomics Proteomics Bioinformatics. 2025 Sep 14;23(5):qzaf083. doi: 10.1093/gpbjnl/qzaf083 (PMC12854720; doi:10.1093/gpbjnl/qzaf083)
Supplement: qzaf083_Supplementary_Data [file qzaf083_supplementary_data.zip › Figure S1.pdf]

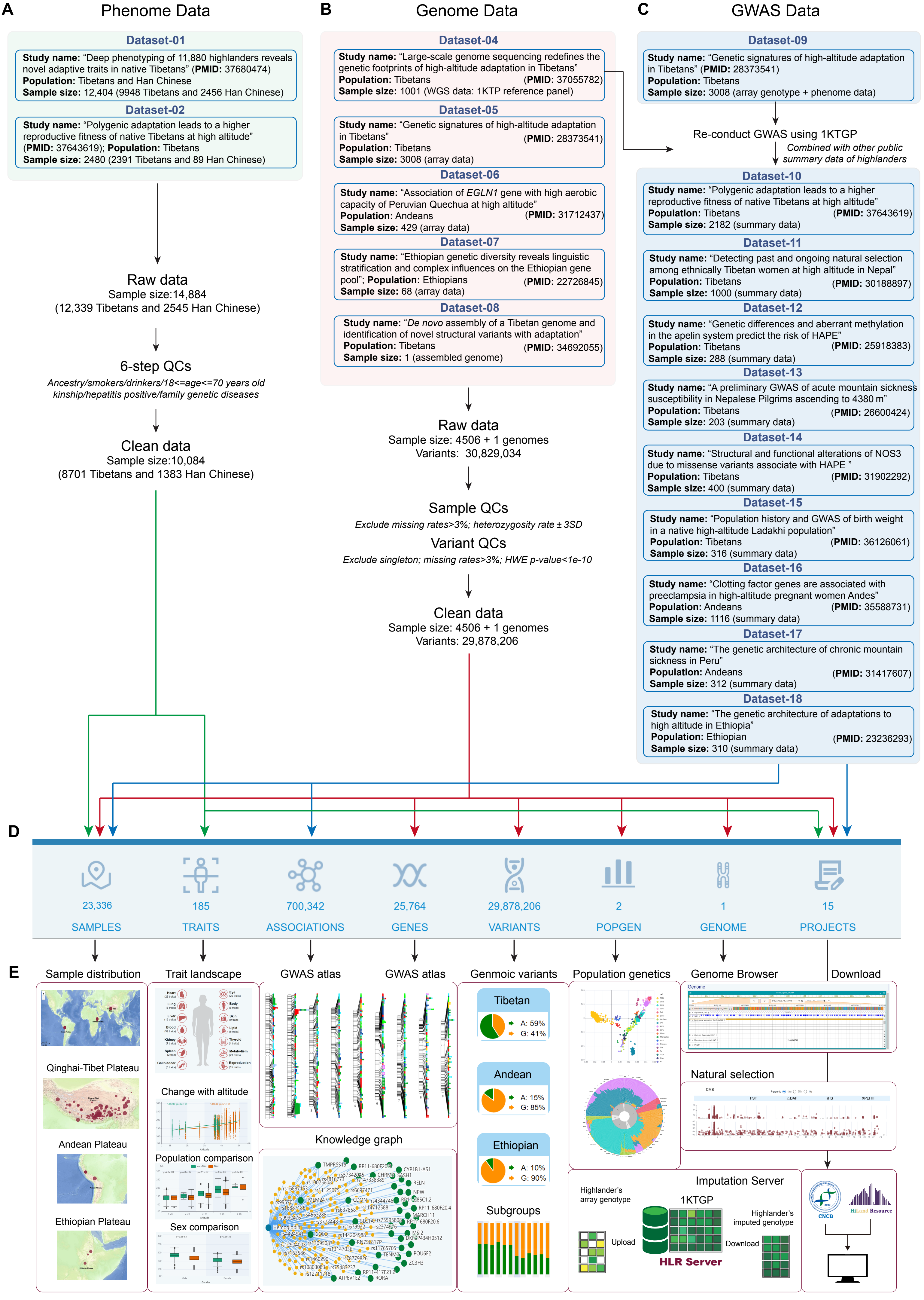

A
Phenome Data
Dataset-01
Study name: "Deep phenotyping of 11,880 highlanders reveals novel adaptive traits in native Tibetans" (PMID: 37680474)
Population: Tibetans and Han Chinese
Sample size: 12,404 (9948 Tibetans and 2456 Han Chinese)
Dataset-02
Study name: "Polygenic adaptation leads to a higher reproductive fitness of native Tibetans at high altitude" (PMID: 37643619); Population: Tibetans
Sample size: 2480 (2391 Tibetans and 89 Han Chinese)
Raw data
Sample size:14,884
(12,339 Tibetans and 2545 Han Chinese)
6-step QCs
Ancestry/smokers/drinkers/18<=age<=70 years old
kinship/hepatitis positive/family genetic diseases
Clean data
Sample size:10,084
(8701 Tibetans and 1383 Han Chinese)
B
Genome Data
Dataset-04
Study name: "Large-scale genome sequencing redefines the genetic footprints of high-altitude adaptation in Tibetans"
Population: Tibetans (PMID: 37055782)
Sample size: 1001 (WGS data: 1KTP reference panel)
Dataset-05
Study name: "Genetic signatures of high-altitude adaptation in Tibetans" (PMID: 28373541)
Population: Tibetans
Sample size: 3008 (array data)
Dataset-06
Study name: "Association of EGLN1 gene with high aerobic capacity of Peruvian Quechua at high altitude"
Population: Andeans (PMID: 31712437)
Sample size: 429 (array data)
Dataset-07
Study name: "Ethiopian genetic diversity reveals linguistic stratification and complex influences on the Ethiopian gene pool"; Population: Ethiopians (PMID: 22726845)
Sample size: 68 (array data)
Dataset-08
Study name: "De novo assembly of a Tibetan genome and identification of novel structural variants with adaptation"
Population: Tibetans (PMID: 34692055)
Sample size: 1 (assembled genome)
Raw data
Sample size: 4506 + 1 genomes
Variants: 30,829,034
Sample QCs
Exclude missing rates>3%; heterozygosity rate ± 3SD
Variant QCs
Exclude singleton; missing rates>3%; HWE p-value<1e-10
Clean data
Sample size: 4506 + 1 genomes
Variants: 29,878,206
C
GWAS Data
Dataset-09
Study name: "Genetic signatures of high-altitude adaptation in Tibetans" (PMID: 28373541)
Population: Tibetans
Sample size: 3008 (array genotype + phenome data)
Re-conduct GWAS using 1KTGP
Combined with other public summary data of highlanders
Dataset-10
Study name: "Polygenic adaptation leads to a higher reproductive fitness of native Tibetans at high altitude"
Population: Tibetans (PMID: 37643619)
Sample size: 2182 (summary data)
Dataset-11
Study name: "Detecting past and ongoing natural selection among ethnically Tibetan women at high altitude in Nepal"
Population: Tibetans (PMID: 30188897)
Sample size: 1000 (summary data)
Dataset-12
Study name: "Genetic differences and aberrant methylation in the apelin system predict the risk of HAPE"
Population: Tibetans (PMID: 25918383)
Sample size: 288 (summary data)
Dataset-13
Study name: "A preliminary GWAS of acute mountain sickness susceptibility in Nepalese Pilgrims ascending to 4380 m"
Population: Tibetans (PMID: 26600424)
Sample size: 203 (summary data)
Dataset-14
Study name: "Structural and functional alterations of NOS3 due to missense variants associate with HAPE"
Population: Tibetans (PMID: 31902292)
Sample size: 400 (summary data)
Dataset-15
Study name: "Population history and GWAS of birth weight in a native high-altitude Ladakhi population"
Population: Tibetans (PMID: 36126061)
Sample size: 316 (summary data)
Dataset-16
Study name: "Clotting factor genes are associated with preeclampsia in high-altitude pregnant women Andes"
Population: Andeans (PMID: 35588731)
Sample size: 1116 (summary data)
Dataset-17
Study name: "The genetic architecture of chronic mountain sickness in Peru"
Population: Andeans (PMID: 31417607)
Sample size: 312 (summary data)
Dataset-18
Study name: "The genetic architecture of adaptations to high altitude in Ethiopia"
Population: Ethiopian (PMID: 23236293)
Sample size: 310 (summary data)
D
23,336 SAMPLES
185 TRAITS
700,342 ASSOCIATIONS
25,764 GENES
29,878,206 VARIANTS
2 POPGEN
1 GENOME
15 PROJECTS
E
Sample distribution
Qinghai-Tibet Plateau
Andean Plateau
Ethiopian Plateau
Trait landscape
Change with altitude
Population comparison
Sex comparison
GWAS atlas
GWAS atlas
Knowledge graph
Genmoic variants
Tibetan
A: 59%
G: 41%
Andean
A: 15%
G: 85%
Ethiopian
A: 10%
G: 90%
Subgroups
Population genetics
Genome Browser
Natural selection
Download
Imputation Server
Highlander's array genotype
Upload
1KTGP
HLR Server
Highlander's imputed genotype
Download
CNCB
HiLand Resource
